# Nuclear Localization of CD26 Induced by a Humanized Monoclonal Antibody Inhibits Tumor Cell Growth by Modulating of POLR2A Transcription

**DOI:** 10.1371/journal.pone.0062304

**Published:** 2013-04-29

**Authors:** Kohji Yamada, Mutsumi Hayashi, Hiroko Madokoro, Hiroko Nishida, Wenlin Du, Kei Ohnuma, Michiie Sakamoto, Chikao Morimoto, Taketo Yamada

**Affiliations:** 1 Department of Pathology, School of Medicine, Keio University, Shinjuku-ku, Tokyo, Japan; 2 Department of Therapy Development and Innovation for Immune Disorders and Cancers, Graduate School of Medicine, Juntendo University, Bunkyo-ku, Tokyo, Japan; Wayne State University, United States of America

## Abstract

CD26 is a type II glycoprotein known as dipeptidyl peptidase IV and has been identified as one of the cell surface markers associated with various types of cancers and a subset of cancer stem cells. Recent studies have suggested that CD26 expression is involved in tumor growth, tumor invasion, and metastasis. The CD26 is shown in an extensive intracellular distribution, ranging from the cell surface to the nucleus. We have previously showed that the humanized anti-CD26 monoclonal antibody (mAb), YS110, exhibits inhibitory effects on various cancers. However, functions of CD26 on cancer cells and molecular mechanisms of impaired tumor growth by YS110 treatment are not well understood. In this study, we demonstrated that the treatment with YS110 induced nuclear translocation of both cell-surface CD26 and YS110 in cancer cells and xenografted tumor. It was shown that the CD26 and YS110 were co-localized in nucleus by immunoelectron microscopic analysis. In response to YS110 treatment, CD26 was translocated into the nucleus via caveolin-dependent endocytosis. It was revealed that the nuclear CD26 interacted with a genomic flanking region of the gene for POLR2A, a subunit of RNA polymerase II, using a chromatin immunoprecipitation assay. This interaction with nuclear CD26 and POLR2A gene consequently led to transcriptional repression of the *POLR2A* gene, resulting in retarded cell proliferation of cancer cells. Furthermore, the impaired nuclear transport of CD26 by treatment with an endocytosis inhibitor or expressions of deletion mutants of CD26 reversed the POLR2A repression induced by YS110 treatment. These findings reveal that the nuclear CD26 functions in the regulation of gene expression and tumor growth, and provide a novel mechanism of mAb-therapy related to inducible translocation of cell-surface target molecule into the nucleus.

## Introduction

CD26 is a type II membrane-spanning glycoprotein that possesses intrinsic dipeptidyl peptidase IV (DPPIV) activity [1], and is implicated in a wide variety of physiological processes, including glucose metabolism, homing and activation of T lymphocytes, and cell adhesion [2], [3]. CD26 has also been identified as one of the cell surface markers associated with various types of cancers and a subset of cancer stem cells in malignant mesothelioma and colorectal cancer [4], [5], [6]. Recent studies have suggested that CD26 expression is involved in tumor growth, tumor invasion, and metastasis [3], [7], [8]. However, the molecular evidence to support such a role for CD26 in cancer cells has been lacking.

We have previously developed anti-CD26 mAbs that exhibit unambiguous inhibitory effects against the growth of cultured cells and xenografted tumors [9], [10]. Notably, the humanized anti-CD26 mAb, YS110, which recognizes the cell membrane-proximal glycosylated region starting at the 20-amino acid flexible stalk region of human CD26, has demonstrated meaningful antitumor effects in malignant mesothelioma models [4]. As it has a human IgG_1_ backbone, YS110 can efficiently mediate the recruitment to tumors of human immune effector cells, including natural killer (NK) cells, that express Fc receptors at the cell membrane, in a process of antibody-dependent cellular cytotoxicity (ADCC) [11], [12]. This Fc domain-based mechanism is commonly observed with other therapeutic mAbs (e.g., trastuzumab and rituximab) [13], [14]. Furthermore, accumulating evidence has shown that these mAbs, which have been approved for cancer therapy, also manifest direct antitumor effects. It has been reported that treatment with trastuzumab, a humanized anti-ErbB2 mAb, reduces the growth of cultured cancer cells by disturbing an associated signaling pathway [11]. In keeping with this, although there is no information on the signaling pathway associated with CD26, YS110 treatment also results in direct inhibitory effects on the proliferation of malignant mesothelioma cells [4]. However, the molecular mechanism underlying this direct inhibitory effect on cell growth, following YS110 treatment of CD26-positive tumors, has yet to be elucidated.

Most cell surface receptors undergo internalization through certain endocytic process. Endocytosis of these receptors has long been thought to be a negative feedback mechanism for regulating receptor function. However, recent evidence has suggested that internalized receptors are involved in signaling functions of the endosome, or directly transmit signals to the nucleus [15], [16]. The latter process is characterized by dynamic nuclear translocation of cargo proteins. Some cell surface receptors, such as epidermal growth factor receptor (EGFR), ErbB2, fibroblast growth factor receptor (FGFR) and CD40, are shown to be translocated into the nucleus, and be consequently involved in transcriptional regulation, cell proliferation, and chemo- and radio-resistance [17], [18], [19]. This emphasizes the significance of nuclear transport of endocytic cargo in the development of strategies for cell surface receptor-targeted therapy.

Nuclear localization of CD26 has been reported in cultured malignant mesothelioma and malignant T cell lines, and in human thyroid carcinomas [20], [21]. However, the functional relevance of nuclear CD26 in cancer cells is far from clear. Our previous studies have shown that the murine anti-CD26 mAb, 1F7, which recognizes the identical epitope to YS110 and has antitumor effects against T cell malignancies, promotes internalization of CD26, and subsequently induces its nuclear accumulation [20], [22]. Therefore, we hypothesized that nuclear localization of CD26 is functionally related to the antitumor process following treatment with YS110. In this study, we demonstrated that nuclear translocation of CD26 and YS110 contributed to growth inhibition of malignant mesothelioma cells after YS110 treatment. By performing chromatin immunoprecipitation (ChIP) cloning, we showed that nuclear CD26 interacted with a specific genome target flanking the gene for POLR2A, which is essential for the transcription of many genes [23]. This interaction led to suppression of POLR2A. Furthermore, blocking the nuclear trafficking of CD26 and YS110 prevented both the nuclear translocation of these two proteins and the YS110-induced transcriptional repression of the POLR2A gene. These findings highlight a novel function of CD26 as a transcriptional modulator in the nucleus, and provide insight into the development of cancer therapies through modulation of the nuclear translocation of cell-surface proteins.

## Materials and Methods

### Cell Culture and Tissue Specimens

The malignant mesothelioma cell lines, JMN, MSTO and MSTO (control, clone 8 and clone 12) [8], the T cell leukemia Jurkat cell line (control and CD26) [22], and the hepatocellular carcinoma Li7 and Kim1 (National Cancer Center Research Institute, Tokyo, Japan) and PLC/PRF/5 (Alex) (American Type culture Collection, Manassas, VA, USA) cell lines were cultured in RPMI 1640 supplemented with 10% fetal bovine serum, 100 units/mL penicillin, and 100 µg/mL streptomycin, under 5% CO_2_ at 37°C. Human embryonic kidney 293 cells and the cervical cancer HeLa cell line were cultured in Dulbecco’s modified Eagle’s medium supplemented with 10% fetal bovine serum, 100 units/mL penicillin, and 100 µg/mL streptomycin, under 5% CO_2_ at 37°C. Malignant mesothelioma specimens from autopsies were generously permitted by the bereaved families. This study was approved by the Keio University School of Medicine review board and the permission was obtained (ID number 2012-100-1). The specimens were subjected to immunostaining as described below. The purpose of the study was explained to all patients and their written, informed consent was obtained.

### Plasmids, siRNA Transfection, and Reporter Assays

pEGFPC1-Rab5A^wt^, pEGFPC1-Rab5A^S34N^, and pEGFPN3-caveolin-1 vectors were a generous gift from Dr. Oikawa (Keio University, Tokyo, Japan). The cDNAs for full-length CD26 (amino acids 1–766), cytoplasmic region-deficient CD26_7–766_ (amino acids 7–766), extracellular region-deleted CD26_1–500_, and CD26_1–629_ were amplified by PCR and introduced into pcDNA3, pFlag-CMV4 (Sigma, St. Louis, MO), and pEGFP-C1 vectors. The PCR primers were: CD26_wt_, 5′-CCG GAA TTC AAT GAA GAC ACC GTGG-3′ and 5′-CGG GAT CCT CAA GGT AAA GAG AAA CA-3′; CD26_7–766_, 5′-CCG GAA TTC AGT TCT TCT GGG ACT GC-3′ and 5′-CGG GAT CCT CAA GGT AAA GAG AAA CA-3′; CD26_1–500_, 5′-CCG GAA TTC AAT GAA GAC ACC GTGG-3′ and 5′-CCG GAA TTC TCA CAA AGC TGA ATTG-3′; and CD26_1–629_, 5′-CCG GAA TTC AAT GAA GAC ACC GTGG-3′ and 5′-CGG GAT CCT CAC CAG CCC CAA ATTG-3′. The resulting vectors were transfected into JMN, HeLa, HEK293, and Li7 cells with Lipofectamine LTX (Invitrogen, Tokyo, Japan). CD26-expressing cells were obtained by selection with G418, as previously described [10].

The sequences of the siRNA (chimeric RNA-DNA) duplexes (RNAi Inc, Tokyo, Japan) were: CHC oligo 1, 5′-CCA AUU CGA AGA CCA AUU UCA-3′ and 5′-AAA UUG GUC UUC GAA UUG GAU-3′; CHC oligo 2, 5′-CUA UGA CAG UCG CGU UGU UGG-3′ and 5′-AAC AAC GCG ACU GUC AUA GUA-3′; caveolin-1, 5′-CCU UCA CUG UGA CGA AAU ACU-3′ and 5′-UAU UUC GUC ACA GUG AAG GUG-3′; POLR2A oligo 1, 5′-CAA CUC CGU ACA AUG CAG ACU-3′ and 5′-UCU GCA UUG UAC GGA GUU GUC-3′; and POLR2A oligo 2, 5′-CAC AUC AAU UGU AUC CGU ACC-3′ and 5′-UAC GGA UAC AAU UGA UGU GAC-3′. Cells were transfected with each siRNA for 48 hours using Oligofectamine reagent (Invitrogen).

For the reporter assays, CAS162 was amplified by PCR and cloned in-frame into pGL3-promoter vector (Promega, Madison, WI). The resulting vector was co-transfected with the phRL-TK vector (Promega). Luciferase assays were performed using a Dual Luciferase Assay Kit (Promega).

### Labeling and Preparation of Antibodies

Human IgG_1_ and YS110 were labeled using an Alexa647 labeling kit (Invitrogen). Mouse IgG_1_ and 1F7 were biotinylated using a biotin labeling kit (Thermo, Rockford, USA).

### Cell Surface Biotinylation and Immunoprecipitation

Cells were washed twice with phosphate-buffered saline (PBS) containing 1 mM CaCl_2_ and 0.5 mM MgCl_2_ (PBS-CM) at 4°C, and incubated for 12 minutes at 4°C in 1.0 mg/mL of sulfo-N-hydroxysulfosuccinimide (sulfo-NHS)-Biotin (Pierce) dissolved in PBS-CM. To quench unreacted biotin, cells were washed three times with PBS-CM plus 50 mM glycine and twice with PBS-CM [24], [25]. After incubation with the indicated antibodies, immunoprecipitation was performed overnight at 4°C, using the CD26 mAb, 1F7 (2 µg), with equal amounts of protein from each fraction. The antibody was bound directly to Protein A Sepharose beads for 1 hour at 4°C. The beads were washed four times with NET-2 buffer (250 mM Tris-HCl pH 7.5, 750 mM NaCl, 0.25% Nonidet P-40) and then subjected to immunoblot analysis [26].

### Histology and Immunohistochemistry

Tissues were fixed in 10% neutral buffered formalin, embedded in paraffin, and sectioned at a thickness of 5 µm. Sections were paraffin depleted and rehydrated in a graded series of ethanol solutions. Alternatively, frozen sections were fixed in 4% paraformaldehyde for 20 minutes at room temperature. For histology, sections were stained with hematoxylin and eosin. For immunohistochemistry, sections were washed with PBS, subjected to antigen retrieval by heating at 100°C in 0.01 M sodium citrate (pH 6.0) for 10 minutes, then treated with 3% H_2_O_2_, before incubation with the following primary antibodies: goat anti-CD26 pAb (AF1180, R&D Systems, Minneapolis, MN) (1∶100), rabbit anti-CD26 pAb (H270, Santa Cruz Biotechnology, Inc, Santa Cruz, CA) (1∶100), and mouse anti-POLR2A mAb (sc-47701, Santa Cruz) (1∶100). Immune complexes were detected by using an ImmPRESS REAGENT KIT (Vector Laboratories, Burlingame, CA) with 3, 3′-diaminobenzidine, and sections were counterstained with hematoxylin.

### Immunoelectron Microscopic Analysis

JMN cells treated with YS110 for 2 hours were fixed in 0.1 M cacodylate buffer (0.1% glutaraldehyde and 4% paraformaldehyde, pH 7.4) on ice overnight. The cells were dehydrated by two 5-minute incubations in 50, 70, 95, and 100% dimethylformamide in water. Cell pellets were incubated in dimethylformamide/lowicryl (1∶1) for 30 minutes at room temperature. Sections (8 nm) were sectioned and mounted on copper mesh with 150 grids, incubated with primary antibodies for CD26 (H270, Santa Cruz) and rabbit anti-early endosome marker (EEA)1 pAb (sc-6415, Santa Cruz) overnight, washed four times with PBS, and labeled for 60 minutes with secondary anti-rabbit antibody conjugated with 15 nm immunogold (GE Healthcare, Uppsala, Sweden) or anti-human F(ab’)_2_ or IgG antibodies conjugated with 30 nm immunogold (GAF-352 and GAF-001, EY Laboratories Inc, San Mateo, CA). Sections were washed with 2% uranyl acetate, followed by 4% lead citrate and visualized by electron microscopy.

### Immunofluorescence Analysis

Tissues and cultured cells grown on glass coverslides were fixed in 4% paraformaldehyde for 20 minutes at room temperature, then permeabilized with PBS containing 0.2% Triton-X-100 and 1 mg/mL bovine serum albumin (BSA) for 25 minutes. The tissues and cells were washed three times with PBS before incubation at 4°C overnight with the following primary antibodies: goat anti-CD26 pAb (AF1180, R&D Systems) (1∶100), rabbit anti-clathrin mAb (610499, BD Pharmingen, San Diego, CA) (1∶100), rabbit anti-caveolin-1 pAb (sc-894, Santa Cruz) (1∶100), and goat anti-EEA1 pAb (sc-6415, Santa Cruz) (1∶200). After washing three times with PBS, the tissues and cells were incubated at room temperature for 30 minutes with the appropriate Alexa Fluor 488-, 594-, or 647-conjugated secondary antibodies and stained with Hoechst 33342 (Invitrogen) for detection of nuclei. Tissues and cells were viewed directly by confocal fluorescence microscopy (FV10i, Olympus, Tokyo, Japan). Quantitation was performed using TissueQuest software (TissueGnostics, Vienna, Austria).

### Tracers and Reagents

Alexa Fluor 488-transferrin, Alexa Fluor 488-cholera toxin B, and fluorescein isothiocyanate (FITC)-dextran were purchased from Invitrogen. Nystatin, filipin, monodansylcadaverine (MDC), and chlorpromazine were obtained from Sigma.

### Subcellular Fractionation and Immunoblot Analysis

Cells were incubated in medium with/without the CD26 mAbs, 1F7 or 5F8 (2 µg/mL), or control mouse IgG_1_ (Dako Cytomation, Glostrup, Denmark). After appropriate incubations, cells were harvested and washed with PBS. Membrane, cytoplasmic and nuclear fractions were extracted using a Qproteome cell compartment kit, according to the manufacturer’s instructions (Qiagen, Hilden, Germany), with minor modifications. The membrane fraction contains endosomes and membrane compartment organelles, such as mitochondria and endoplasmic reticulum (ER), as well as cell surface proteins. Alternatively, two cytoplasmic and nuclear fractions were extracted using NE-PER Nuclear and Cytoplasmic Extraction Reagents (Pierce, Thermo Fisher Scientific, Rockford, IL), with minor modifications. The protein concentrations of each fraction were determined using the bicinchoninic acid (BCA) protein assay kit (Pierce Biotechnology). Equal amounts of protein were subjected to 10% sodium dodecyl sulfate (SDS) polyacrylamide gel electrophoresis (PAGE) and transferred to a polyvinylidene fluoride (PVDF) membrane. The membranes were probed with the following antibodies: human CD26 with goat polyclonal antibody (pAb) (AF1180, R&D Systems) (1∶200), human POLR2A with mouse mAb (sc-47701, Santa Cruz) (1∶200), Na^+^/K^+^ ATPase as a plasma membrane marker with mouse mAb (sc-21712, Santa Cruz) (1∶1000), culreticulin as an ER marker with mouse mAb (612136, BD Pharmingen) (1∶2000), calpain-1/2 as a cytoplasmic marker with mouse mAb (Calbiochem, La Jolla, CA) (1∶2000), Lamin A/C as a nuclear marker with mouse mAb (sc-7292, Santa Cruz) (1∶200), and nucleostemin as a nuclear marker with mouse mAb (Qiagen) (1∶1000). Signals were detected by enhanced chemiluminescence (ECL). The relative amounts of CD26 expressed in representative experiments were quantitatively analyzed using ImageQuant 350 software (GE Healthcare), and indicated as the percentage CD26 expression in each fraction.

### Chromatin Immunoprecipitation Assay

Chromatin immunoprecipitation was performed using a Simple ChIP Kit (Cell Signaling Technology, Tokyo, Japan), according to the manufacturer’s instructions. Cells treated with control human IgG_1_ or YS110 were fixed in 1% formaldehyde, and sonicated. After centrifugation, the supernatants containing immunocomplexes were incubated with anti-human IgG_1_ or goat anti-CD26 pAb (AF1180, R&D Systems) at 4°C overnight, and then for a further 2 hours with protein G-conjugated magnetic dynabeads. After the immunocomplexes were washed six times with washing buffer, DNA was reverse cross-linked by incubation at 65°C for 2 hours and used for cloning, or as a template for PCR. The identified CAS162 sequence was: 5′-AGC TGA AGT AAA AGG ACT TGG GGG TAA TAC GCT AGT TTT AGC CGG CTA TTT TTC CCC CTT TCA TTA GCA CCT TAA TGT GGT ATC AAT GTT CTA CAT CCT CTG CAA GTC ATT TCT GAT TTA CCT GAG GTA-3′. The PCR primer sequences for CAS162 were: 5′-AGC TGA AGT AAA AGG ACT TGG-3′ and 5′-TAC CTC AGG TAA ATC AGA AAT GAC-3′.

### Cloning of DNA Fragments

Immunoprecipitated DNA fragments were cloned into the PCRII Blunt TOPO Vector (Invitrogen). Each DNA sequences were searched using Blast analysis and the National Institutes of Health Entrez Genome Project database.

### Electrophoretic Mobility Shift Assay (EMSA)

Double-stranded oligonucleotides containing the 129-bp CD26-associated sequence (CAS) 162 were labeled with biotin using a Biotin 3′ End DNA Labeling Kit (Thermo Fisher Scientific). Nuclear extracts were prepared from JMN cells treated with YS110 (2 µg/mL) for 2 hours, using the NE-PER Nuclear and Cytoplasmic Extraction Reagent Kit (Thermo Fisher Scientific). EMSA was performed using the LightShift Chemiluminescent EMSA Kit (Thermo Fisher Scientific) according to the manufacturer’s instructions, with minor modifications. The reaction products were separated by electrophoresis in a 5% non-denaturing polyacrylamide gel (Invitrogen) in 0.5% TB. In competition experiments, nuclear extracts were pre-incubated with a 100-fold excess of intact competitive CAS162 oligonucleotides before biotin-labeled CAS162 was added.

### Quantitative RT-PCR Analysis

Total RNA was extracted from cultured cells with RNeasy mini kits (Qiagen) according to the manufacturer’s instructions. Reverse transcription of purified RNA was performed using a PrimeScript RT-PCR kit (Takara Bio Inc, Shiga, Japan), according to the manufacturer’s instructions. Quantification of all gene transcripts was performed by qPCR, using SYBR Premix Ex Taq II and a Thermal Cycler Dice Real Time System (Takara). The primer pairs were: POLR2A, 5′-GCA TGG CAG AGG AGT TTC GGCT-3′ and 5′-ATT TCC CCG GGA TGC GCA ATGG-3′; and GAPDH, 5′-CCA GCC GAG CCA CAT CGC TC-3′ and 5′-ATG AGC CCC AGC CTT CTC CAT-3′.

### Proliferation Assay

Cells were incubated in 96-well plates in media alone or with CD26 mAb (0.02, 0.2, 2, or 20 µg/mL) in a total volume of 100 µL (2.5×10^3^ cells/well). After 24 or 48 hours of incubation at 37°C, 2-(2-methoxy-4-nitrophenyl)-3-(4-nitrophenyl)-5-(2,4-disulfophenyl)-2H-tetrazolium (WST) (Nacalai Tesque Inc, Tokyo, Japan) was added to each well. After a further 1 hour of incubation, the water-soluble formazan dye, 1-methoxy-5-methylphenazinium, formed upon bio-reduction in the presence of an electron carrier, was measured in a microplate reader (Bio-Rad) at 450 nm. All samples were assayed in triplicate, and the results reported were means of triplicate wells.

### Xenograft Model Using Human Mesothelioma Cell Lines

NOD/Shi-scid, IL-2 receptor gamma null (NOG) mice were obtained from the Central Institute for Experimental Animals. JMN cells (1×10^6^) were implanted subcutaneously in the back flank of NOG mice. Mice were injected intratumorally with control human IgG_1_ (n = 3) or YS110 (n = 3) at doses of 8 mg/kg body weight. Parental MSTO cells or MSTO cells stably expressing CD26 were inoculated into the thoracic cavities of NOG mice. Thereafter, mice were intraperitoneally injected with control human IgG_1_ (n = 3), or YS110 (10 µg per injection) (n = 3), commencing on the day of cancer cell injection. Each antibody was administered three times per week. Mice were then monitored for the development and progression of tumors. Tumor size was determined by caliper measurement of the largest (*x*) and smallest (*y*) perpendicular diameters, and was calculated according to the formula *V* = *π*/6×*xy* ^2^.

All experiments were approved by the Animal Care and Use Committee of Keio University and were performed in accordance with the institute guidelines.

### Statistical Analysis

Data are presented as means ± SD and were assessed for statistical significance using the unpaired Student’s t test.

## Results

### Anti-CD26 Monoclonal Antibodies are Translocated to the Nucleus in CD26-Positive Cancer Cells

**Figure 1:**
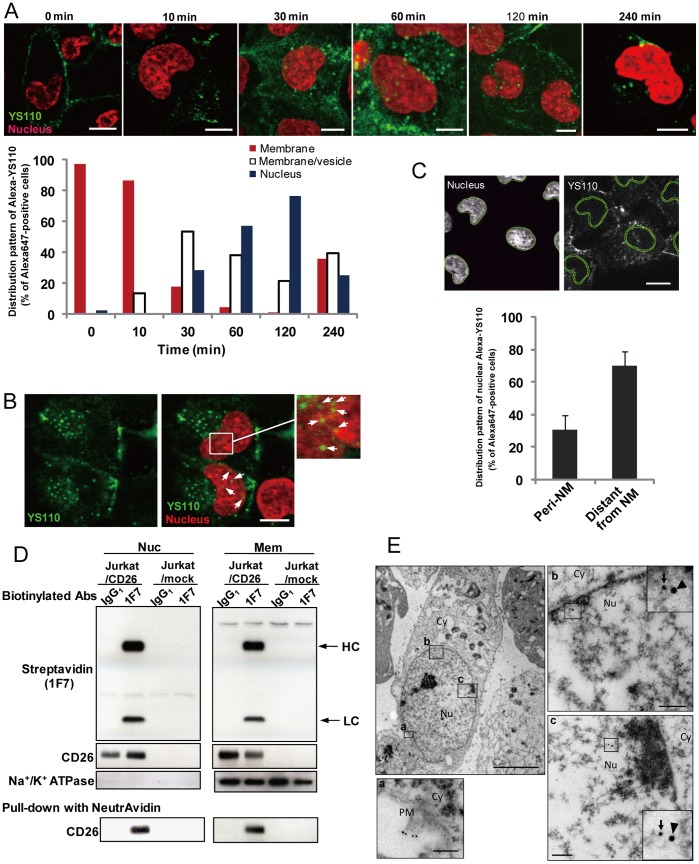
Nuclear Translocation of Antitumor CD26 mAbs in Cancer Cells. (A) JMN cells were treated with Alexa647-labeled YS110 (Alexa-YS110) for the indicated time periods before fixation. The distribution of Alexa-YS110 in the fixed cells was observed by confocal fluorescence microscopy (upper panels). To quantitate these observations, fixed JMN cells retaining Alexa-YS110 were categorized into three types [28]: the cell containing YS110 predominantly observed on the cell surface (red bar); the cell containing Alexa-YS110 present on both the cell surface and in cytosolic vesicles (white bar); the cell containing Alexa-YS110 observed in the nucleus (blue bar). The categorization was performed by confocal fluorescence microscopy of more than 50 cells for each incubation time (lower panel). Scale bars, 10 µm. (B) Immunofluorescence staining with antibody to human IgG_1_ in fixed JMN cells, following YS110 treatment for 1 hour, and staining with Hoechst 33342. Localization of YS110 (green) in the nucleus (red) appears as yellow, as indicated by arrows. Scale bars, 10 µm. (C) Identification of the nuclear membrane (NM) was performed using TissueQuest software. The distribution of Alexa-YS110 in the nucleus was subdivided into two categories: close to (within 1 µm; peri-NM) or distant from NM, in JMN cells treated with Alexa-YS110 for 2 hours. Data are means ± SD for more than 20 cells. Scale bar, 10 µm. (D) Jurkat/mock or Jurkat/CD26 cells were incubated with biotin-labeled control IgG_1_ or 1F7 for 1 hour. Nuclear (Nuc) and membrane (Mem) extracts of these cells were pulled-down with Neutravidin, and then subjected to immunoblot analysis using streptavidin or antibodies to CD26 or Na^+^/K^+^ ATPase (membrane marker). HC, heavy chain; LC, light chain. (E) Immunogold labeling for CD26 and YS110 on ultrathin sections demonstrated the localization of these proteins in JMN cells. The arrow and arrowhead indicate CD26 (15 nm) and YS110 (30 nm) in the plasma membrane (a) and the nucleus (b and C), respectively. Scale bars, 5 µm and 200 nm (a, b and c). PM, plasma membrane; Cy, cytoplasm; Nu, nucleus.

The cellular localization of YS110 in malignant mesothelioma cells was examined to elucidate the role of humanized mAb YS110 in its antitumor effect on cancer cells. The CD26-positive malignant mesothelioma cell line, JMN (the proliferation of which was reduced after YS110 treatment) (Fig. S1A), was chosen, and the cells were treated with YS110 labeled with Alexa647 dye (Alexa-YS110). After treatment, Alexa-YS110 was internalized and diffused throughout the cytoplasm within 30 min (Fig. 1A). Furthermore, at 30 min to 2 h, Alexa-YS110 localized within the nucleus in the form of dots, as well as in the cytoplasm (Fig. 1A). This observation of several dots of Alexa-YS110 that colocalized with a nuclear marker (Hoechst 33342) in a single cell defined the cells containing YS110 in the nucleus. Over 4 h after treatment with Alexa-YS110, the number of cells retaining Alexa-YS110 in the nucleus was apparently decreased (Fig. 1A). To exclude the possibility that this nuclear localization of Alexa647 fluorescence was due to free Alexa647 fluorescent probe that had detached from YS110, indirect immunostaining analysis was performed with anti-human IgG. Nuclear staining of YS110 was apparent in JMN cells treated with unlabeled YS110 prior to fixation (Fig. 1B). Furthermore, on closer examination of YS110 nuclear localization using TissueQuest software [27], it was estimated that about 70% of the nuclear YS110 resided away from the nuclear membrane (NM), with the remaining YS110 distributed diffusely along the perimeter of the inner NM (Fig. 1C). These results suggested that YS110 appears to localize in the nucleus.

To confirm the nuclear localization of this antibody, biochemical analysis was performed using a T cell leukemia cell line (Jurkat) that is negative for CD26, and a Jurkat cell line that was stably transfected with CD26 expression vector (Jurkat/CD26). These cells were treated with biotin-conjugated control IgG_1_ (biotin-IgG_1_), or with biotin-conjugated murine anti-CD26 mAb, 1F7 (biotin-1F7), which recognizes an epitope identical to that recognized by YS110 and has an antitumor effect on T cell malignancies (Fig. S1B) [10]. Upon treatment with biotin-1F7, two biotin bands comparable in size to the heavy and light chains of 1F7 were detected in the nuclear fraction of Jurkat/CD26 cells, whereas no bands were detected in any fraction from control Jurkat/mock cells (Fig. 1D), indicating that 1F7 was localized in the nuclear fraction of CD26-positive Jurkat cells. Furthermore, a pull-down assay with streptavidin demonstrated the association of biotin-1F7 with CD26 in the nuclear fraction of biotin-1F7-treated Jurkat/CD26 cells, but not in cells treated with biotin-IgG_1_ (Fig. 1D, lower panels). Similarly, co-localization of CD26 and YS110 was also observed by immunoelectron microscopic analysis using different sized immunogold particles (15 nm for CD26, 30 nm for YS110) (Fig. 1E). Taken together, these results suggested that two anti-CD26 mAbs (YS110 and 1F7) with antitumor effects on cancer cells are transported to the nucleus in a CD26-dependent manner.

### YS110 Induces Nuclear Localization of CD26, and Deletion of the Extracellular Region of CD26 Prevents the Nuclear Transport of CD26 and YS110

**Figure 2:**
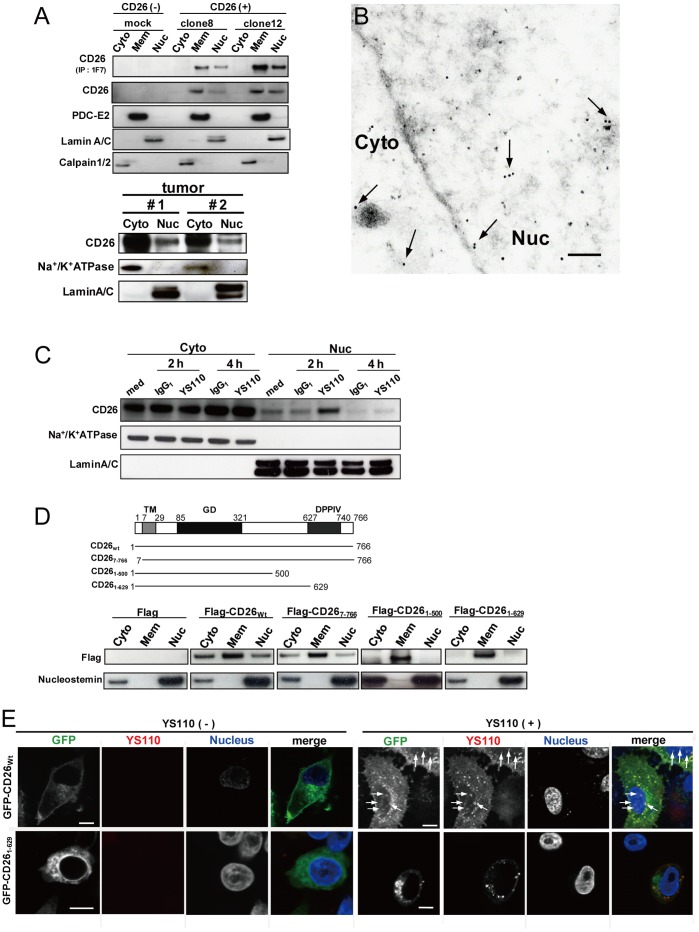
Anti-CD26 mAbs Enhance Nuclear Localization of CD26. (A) Nuclear (Nuc), cytoplasmic (Cyto), and membrane (Mem) fractions of MSTO cells stably transfected with empty vector (mock) or CD26 (clone8 and clone12) were prepared (Qiagen kit), immunoprecipitated with 1F7, and subjected to immunoblot analysis. Nuclear (Nuc) and cytosolic (Cyto) fractions of tumors from two malignant mesothelioma patients were prepared (Thermo kit), and subjected to immunoblot analysis. Lamin A/C, Calpain 1/2, and PDC-E2 and Na^+^/K^+^ ATPase were used as nuclear, cytoplasmic and membrane markers, respectively. (B) Immunogold staining of CD26 (15 nm gold particles, arrows) in ultrathin sections of JMN cells. Cyto, cytoplasm; Nu, nucleus. Scale bar, 200 nm. (C) Immunoblot analysis of CD26 in nuclear and cytosolic fractions of JMN cells treated with YS110 for the indicated times. Na^+^/K^+^ ATPase and Lamin A/C were used as cytosolic and the nuclear markers, respectively. (D) Diagram of each CD26 deletion mutant (left picture). CD26 contains a cytoplasmic domain (amino acids 1–6), a transmembrane domain (TM) (amino acids 7–29), a glycosylated domain (GD) (amino acids 85–321), and a dipeptidyl peptidase IV domain (DPPIV) (amino acids 627–740). Human embryonic kidney (HEK) 293 cells transiently expressing each flag-tagged construct were subjected to subcellular fractionation and immunoblot analysis with antibodies to Flag and nucleostemin (as a nuclear marker). (E) Immunofluorescence analysis of HeLa cells transfected with GFP-CD26_wt_ or GFP-CD26_1–629_ and treated or not treated with Alexa-YS110 for 1 hour. In the merged image, GFP-fused proteins are shown in green, Alexa-YS110 is shown in red, and the nucleus is shown in blue. Arrows indicate colocalization of Alexa-YS110 and CD26_wt_ in the nucleus. Scale bars, 10 µm.

To investigate the nuclear localization of CD26 in cancer cells, four malignant mesothelioma cell lines that differed in CD26 expression status were first examined: one that expressed CD26 endogenously (JMN); one CD26-negative cell line (MSTO); and two cell lines that expressed CD26 exogenously (MSTO/clone8, MSTO/clone12). Under normal culture conditions, the single full-length form of CD26 was detected, not only in the membrane and cytoplasmic fractions, but also in the nuclear fraction of JMN [20], MSTO/clone8 and MSTO/clone12 (MSTO/CD26) cells (Fig. 2A). Similar result was obtained in primary tumor of two malignant mesothelioma patients (Fig. 2A). Furthermore, nuclear localization of CD26 in JMN cells was confirmed by immunoelectron microscopy (Fig. 2B). These results suggested that full-length CD26 is translocated to the nucleus, as previously reported [20].

We next investigated the potential effect of YS110 on nuclear localization of CD26 in cancer cells. Western blot analysis of JMN cells showed that the amount of CD26 in the nuclear fraction of the cells was markedly increased by YS110 treatment, peaking at 2 h and then decreasing by 4 h to close to the original level (Fig. 2C). Similar results were obtained after 1F7 treatment of Jurkat/CD26 cells (Fig. S2A), and after YS110 treatment of Li7 hepatocellular carcinoma cells that expressed CD26 exogenously (Fig. S2B), suggesting that YS110 and 1F7 induced nuclear translocation of CD26 in cancer cells.

To evaluate the relevance of the nuclear localization of CD26 to the nuclear trafficking of YS110, various CD26 deletion mutants were generated to specify the domain of CD26 that was essential for its nuclear localization (Fig. 2D). When transfected into human embryonic kidney (HEK) 293 cells that are negative for CD26, the wild-type (CD26_wt_) and cytoplasmic region-deficient (CD26_7–763_) forms were clearly detected in the nuclear fraction of these cells (Fig. 2D). In contrast, the two partial C-terminal extracellular region-depleted forms (CD26_1–500_ and CD26_1–629_) were not detected in the nuclear fraction, although their cell-surface expression and capacity to bind to YS110 were confirmed (Fig. 2D; Fig. S3A). Similar results were obtained in Li7 cells transfected with CD26_wt_ or CD26_1–629_ constructs (Fig. S2B), indicating that the extracellular domain of CD26 is required for its nuclear transport.

Next, the nuclear trafficking of each CD26 construct and YS110 was examined after YS110 treatment of CD26_wt_ or CD26_1–629_-expressing JMN or HeLa cells, which express higher levels of caveolin-1 than HEK 293 cells [28]. After YS110 treatment of these two GFP-fused CD26_wt_-expressing cell lines, GFP-CD26_wt_ was visible in the nucleus, where it overlapped with Alexa-YS110 fluorescence (Fig. 2E; Fig. S3B). In contrast, no nuclear localization of GFP-CD26_1–629_ and Alexa-YS110 was observed when these cells were transfected with GFP-CD26_1–629_ (Fig. 2E; Fig. S3B). These results suggest that the nuclear trafficking machinery for CD26 mediates the nuclear entry of YS110.

As the nuclear translocation of YS110 and 1F7 appears to rely on the cell-surface expression of CD26 (Fig. 1D), the involvement of cell-surface CD26 in augmented nuclear localization of CD26 by these antibodies was investigated. A cell-surface biotinylation assay using Jurkat/CD26 cells showed that, relative to control, the amount of biotin-labeled CD26 in the nuclear fraction was significantly increased following 1F7 treatment, over the same time course as that of the total CD26 content of the cells after 1F7 treatment (Fig. S2A; S4A), indicating that 1F7 promoted nuclear translocation of cell-surface CD26. Conversely, this phenotype was not seen in cell-surface biotinylated cells treated with another murine anti-CD26 mAb, 5F8, which recognizes a different epitope of CD26 from that recognized by YS110 and 1F7, and which has no antitumor effect on cell growth (Fig. S4B) [22]. These data suggest that antitumor mAbs (YS110 and 1F7) bind to cell-surface CD26, and thereafter are translocated to the nucleus.

### Caveolin-Dependent Endocytosis Affects both Endocytosis and Nuclear Translocation of CD26 and YS110

**Figure 3:**
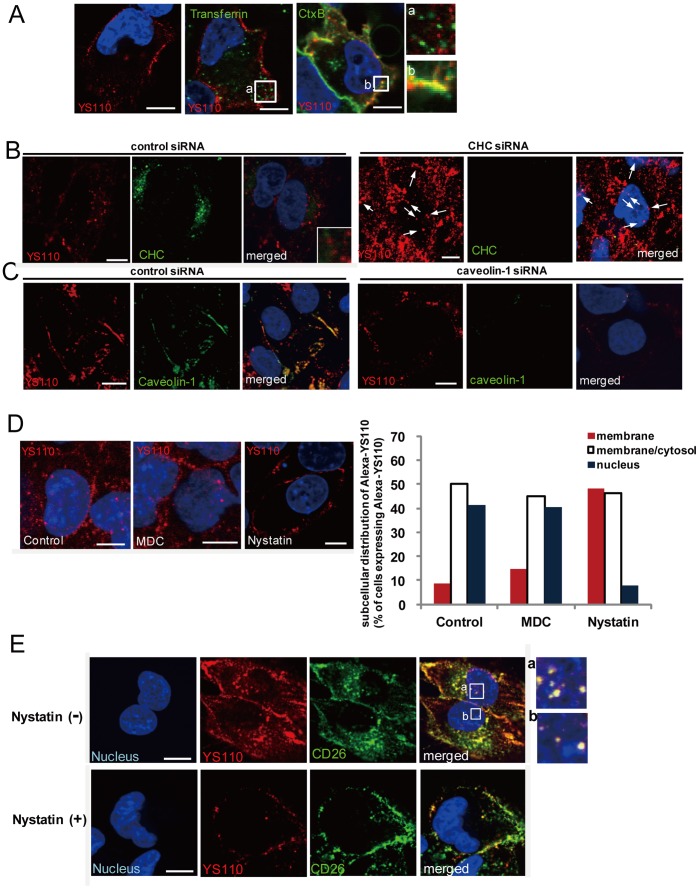
Caveolin-Dependent Endocytosis Mediates the Nuclear Translocation of CD26 and YS110. (A) JMN cells were incubated with Alexa-YS110 and PBS, Alexa488-Transferrin (Alexa-Tf), or Alexa488-Cholera toxin B (Alexa-CtxB) for 5 minutes In the merged images, YS110 is shown in red, the tracers are shown in green, and the nucleus is shown in blue. Colocalization of YS110 and the respective tracer appears as yellow. The boxed region in the panels shows localization of Alexa-YS110 and Alexa-Tf (a) or Alexa-CtxB (b) at high magnification. Scale bars, 10 µm. (B) JMN cells were treated with siRNA for non-silencing (NS) or siRNA for clathrin heavy chain (CHC). Images show immunofluorescence staining for YS110 (red), clathrin heavy chain (CHC, green) and Hoechst 33342 (blue) in fixed JMN cells, following Alexa-YS110 treatment for 30 minutes. Scale bars, 10 µm. (C) JMN cells were treated with siRNA for NS or siRNA for caveolin-1. Images show immunofluorescence staining for YS110 (red), caveolin-1 (green) and Hoechst 33342 (blue) in fixed JMN cells, following Alexa-YS110 treatment for 30 minutes. Scale bars, 10 µm. (D) JMN cells were pre-treated with dimethyl sulfoxide (DMSO), monodansylcadaverin (MDC) (250 µM), or nystatin (50 µg/mL) for 30 minutes, and then stimulated with Alexa-YS110 for 30 minutes. Quantification of the number of cells in which Alexa-YS110 was localized on the cell surface (membrane), in the cytosol (membrane/cytosol), and in the nucleus (nucleus), was performed by confocal fluorescence microscopy of more than 50 cells for each incubation time (right panel). Scale bars, 10 µm. (E) JMN cells were pretreated with or without nystatin prior to incubation with Alexa-YS110 for 30 minutes. In the merged images, YS110 is shown in red, CD26 is shown in green, and the nucleus is shown in blue. Colocalization of YS110 and CD26 in the nucleus appears as white in the boxed region (a and b). Scale bars, 10 µm.

To further characterize the nuclear transport of CD26 and YS110, the involvement of endocytosis in the nuclear translocation of CD26 and YS110 was examined. The process of internalization of cell-surface proteins has been divided into three major mechanisms: clathrin-mediated endocytosis, caveolin-dependent endocytosis, and macropinocytosis [29]. Therefore, we investigated which pathway is used by YS110 to enter the cytosol after binding to cell-surface CD26. To this end, three different endocytotic tracers were employed: Alexa488-labeled transferrin (Alexa-Tf) for the clathrin pathway, Alexa488-labeled cholera toxin B (Alexa-CtxB) for the caveolin pathway, and FITC-dextran for macropinocytosis [30]. Colocalization of Alexa-YS110 was observed throughout the cytoplasm of JMN cells after co-treatment with Alexa-CtxB, but not Alexa-Tf (Fig. 3A; Fig. S5A). While, FITC-dextran was not observed on JMN cells at any time, possibly due to the low reactivity of the cell-surface components with dextran (data not shown). Furthermore, consistent with previous evidence that cell-surface CD26 associates with caveolin-1 at the lipid/raft domain [31], YS110 was found to colocalize with caveolin-1, but not with clathrin heavy chain (CHC), which is a key component of the clathrin pathway (Fig. 3B, 3C). These observations suggest that YS110 may utilize caveolin-dependent endocytosis to enter the nucleus.

In turn, the significance of the caveolin-dependent pathway for the nuclear translocation of CD26 and YS110 was investigated. When JMN cells were depleted of caveolin-1 by transfection with small interfering RNA (siRNA) for caveolin-1 mRNA, significant reductions in both endocytosis and nuclear localization of YS110 were observed in JMN cells treated with Alexa-YS110 (Fig. 3C). However, there was no significant difference in the distribution of YS110 between cells treated with control siRNA or siRNA for CHC (Fig. 3B, arrows). Accordingly, disruption of caveolae formation with nystatin, an inhibitor of the caveolin pathway that binds to cholesterol, markedly impaired both the endocytosis and nuclear localization of CD26 and YS110 in YS110-treated cells (Fig. 3D, 3E). In contrast, two inhibitors of clathrin-mediated endocytosis, monodansylcadaverine (MDC) and chlorpromazine, did not affect the endocytosis and nuclear localization of YS110 (Fig. 3D; Fig. S5B). These results indicated that caveolin-dependent endocytosis is required for the nuclear translocation of CD26 and YS110.

Endocytic transport is regularly exerted by Rab small G proteins [32], [33]. Rab5A organizes a membrane domain that defines the site of entry into early endosomes through its effector proteins, including early endosome antigen (EEA)1. Previous studies have indicated that EEA1 associates with EGFR and ErbB2 in the nucleus [34], [35]. Therefore, we examined whether the nuclear trafficking of YS110 involved an early endocytic pathway. Immunostaining with EEA1 antibody showed that Alexa-YS110 colocalized with EEA1 in the nucleus within 30 min (Fig. S5C, lower panels). YS110-EEA1 complex in the nucleus was also observed by electron microscopy with the respective immunogold particle-conjugated antibodies (15 nm for EEA1, 30 nm for YS110) (Fig. S5D). Importantly, expression of the dominant-negative form of Rab5A (Rab5A^S34N^) suppressed nuclear translocation of Alexa-YS110 (Fig. S5E). These data therefore strongly support the significance of endocytic trafficking in the nuclear translocation of YS110.

### Nuclear Localization of CD26 and YS110 in a Xenograft Model for Malignant Mesothelioma

**Figure 4:**
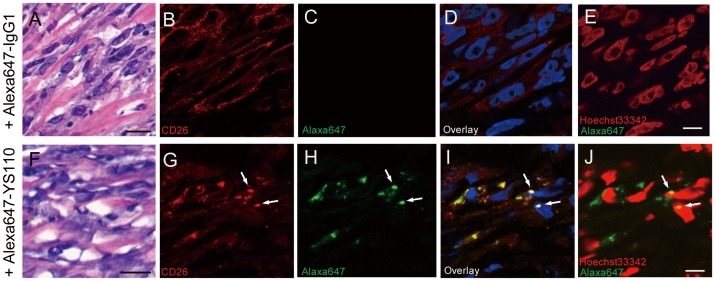
YS110 and CD26 Translocate to the Nucleus *In Vivo*. H&E staining (A and F) and fluorescence analysis (B–E and G–J) of malignant mesothelioma tumors in NOG mice inoculated with JMN cells were shown. These tumors were removed 6 hours after one intratumoral injection (1 µg/a tumor, volume is 100 µL) of Alexa647-human IgG_1_ (A–E) or Alexa647-YS110 (F–J). In the overlaid image, CD26 expression is indicated in red (B, D, G, and I), Alexa647-labeled antibodies are shown in green (C, D, H and I), and the nucleus is shown in blue (D and I). Colocalization of CD26 and YS110 in the nucleus appears as white (I, arrows). This localization of Alexa647-labeled antibody (green) in the nucleus (red) is confirmed as yellow (arrows) (J). Similar results were obtained with three different mice. Scale bars, 20 µm (A and F) and 10 µm (B–E and G–J).

To evaluate whether CD26 and YS110 are translocated to the nucleus *in vivo*, a xenograft model was established using NOD/Shi-scid, IL-2 receptor gamma null (NOG) mice that constitutively lack T, B, and NK cell activities, and were subcutaneously inoculated with JMN cells. The tumors were allowed to develop in the xenografted mice for about two months after inoculation, and exhibited sarcomatoid malignant mesothelioma-like histology (Fig. 4A, 4F). In a similar model, we confirmed that administration of YS110 apparently reduced the tumorigenicity of JMN [4] and MSTO/clone12 (MSTO/CD26) cells (Fig. S6). Direct intra-tumoral injection of Alexa-YS110 into the center of the JMN tumors resulted in the nuclear accumulation of Alexa-YS110, as observed in tumor sections from the mice 1 h (Fig. S7) and 6 h (Fig. 4H, 4I, 4J) after Alexa-YS110 injection. Conversely, there was no Alexa647-fluorescence at any tumor sites injected with Alexa-control IgG_1_ (Fig. 4C, 4D, 4E; Fig. S7). Furthermore, CD26 also localized as vesicle-like structures in the cytosol and nucleus after Alexa-YS110 administration (Fig. 4G, 4I), whereas it was located at the cell surface and in the cytoplasm in tumors treated with Alexa-IgG_1_ (Fig. 4B, 4D). From these results, we conclude that CD26 and YS110 are translocated to the nucleus of CD26-positive cancer cells as a result of treatment with YS110.

### Nuclear CD26 Associates with Target Genomes and Represses POLR2A Gene Expression

**Figure 5:**
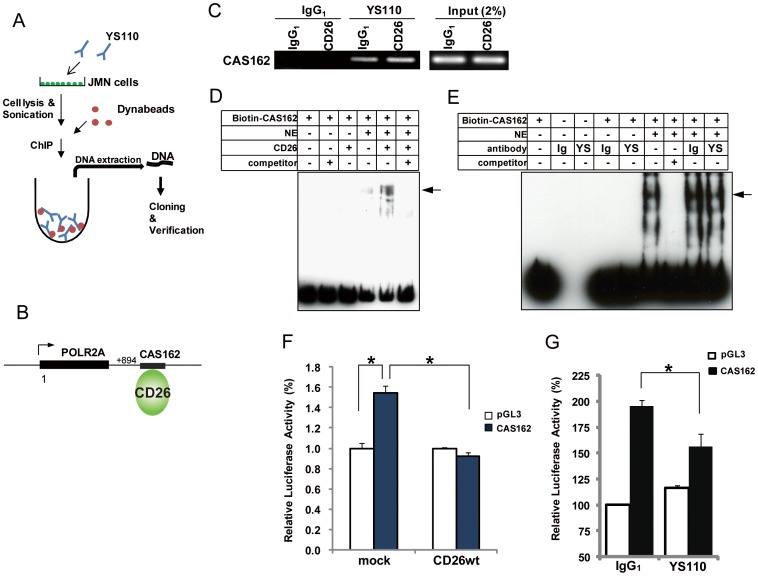
Nuclear CD26 Associates with a POLR2A-Related Genome Sequence Following YS110 Treatment. (A) Schematic diagram of chromatin immunoprecipitation (ChIP) cloning. JMN cells pretreated with YS110 for 3 hours were fixed, sonicated, and immunoprecipitated with Dynabeads to collect YS110-CD26-DNA complexes. The DNA fragments were cloned and identified by sequencing. The identity of candidate sequences was confirmed using data from GeneBank. (B) Genomic location of the CAS162 sequence. The 129-bp CAS162 sequence is located 894 bp downstream from the POLR2A gene. (C) ChIP analysis of CAS162 in JMN cells treated with control human IgG_1_ or YS110 for 3 hours. Similar results were obtained in three independent experiments. (D) Biotin-labeled CAS162 oligonucleotide was used for electrophoretic mobility shift assay (EMSA). Nuclear extract (NE) was also collected from JMN cells pretreated with YS110 for 3 hours. After 20 minutes at room temperature the extracts, with or without recombinant CD26, were subjected to immunoblot analysis with streptavidin. Non-biotinylated CAS162 was used as a competitor. Arrow indicates the CD26-CAS162 oligonucleotide complex. (E) Biotin-labeled CAS162 oligonucleotide was used for EMSA. NE was collected from JMN cells. After 20 minutes at room temperature the extracts, with or without antibodies, were subjected to immunoblot analysis with streptavidin. Arrow indicates the CD26-CAS162 oligonucleotide complex. YS, YS110; Ig, IgG_1_. (F) MSTO (mock or CD26_wt_) cells were co-transfected with empty vector (pGL3) or CAS162 and phRL-TK, and relative luciferase activity was measured using a luminometer. Data were normalized for luciferase activity in cells transfected with phRL-TK, and are presented as mean values (± SD) from three independent experiments. *P<0.025. (G) JMN cells co-transfected with pGL3 or CAS162 and phRL-TK vector were incubated with control IgG_1_ or YS110, and relative luciferase activity was measured using a luminometer. Data were normalized for luciferase activity in JMN cells transfected with phRL-TK, and are presented as mean values (± SD) from three independent experiments. *P<0.025.

To address the potential role of nuclear CD26 in response to YS110 treatment, chromatin immunoprecipitation (ChIP) cloning was performed to explore the possibility that nuclear CD26 associates with genomic targets as a transcriptional regulator. Putative associated DNA fragments were collected from lysates of JMN cells stimulated with YS110 for 3 h, and then identified as candidate sequences, with the loci within 3.0 kilobase pairs (kbp) of genes and introns being identified as nuclear CD26-associated sequences (CAS) (Fig. 5A). Among these, we focused on the CAS162 sequence, which is located 894 bp downstream of the gene that encodes polymerase (RNA) II (DNA directed) polypeptide A (POLR2A; NM_000937) (Fig. 5B). POLR2A is the largest subunit of RNA polymerase II, which is essential for the transcription of most protein-coding genes [23]. Therefore, further investigation was carried out on the relationship between CAS162 and CD26. ChIP analysis using primers flanking CAS162 revealed that the interaction between CD26 and CAS162 was dramatically increased in JMN cells treated with YS110, as compared to those treated with control IgG_1_ (Fig. 5C; Fig. S8A). The small amount of PCR product in control goat IgG-immunoprecipitant from the YS110-treated cells was likely due to the binding of YS110 to protein G sepharose on the dynabeads (Fig. 5C). The association between CD26 and the CAS162 sequence was confirmed by electrophoretic mobility shift assay (EMSA). A shifted complex with biotinylated 129 bp CAS162 was apparently enhanced when recombinant CD26 was added to nuclear extracts of JMN cells (Fig. 5D, arrow). On the other hand, additional YS110 did not affect any shifts or the amount of biotinylated-CAS162 (Fig. 5E, arrow), indicating that CD26 plays a key role in the assembly of the CD26/YS110/CAS162 complex.

To evaluate the transactivation potential of CAS162 in cancer cells, luciferase assays were performed in several cancer cell lines. In contrast to transfection with control vector, transfection with CAS162 reporter significantly increased luciferase activity in several cancer cell lines: two malignant mesothelioma lines (MSTO, JMN); and two hepatocellular carcinoma cell lines (Li7, Kim1) (Fig. 5F, 5G; Fig. S8B), suggesting that CAS162 may contain the regulatory element for POLR2A gene expression. Furthermore, we investigated whether nuclear localization of CD26 would affect this CAS162-regulating luciferase activity. The luciferase activity was significantly reduced in CAS162 reporter-expressing MSTO/CD26 cells, in which CD26 is physiologically localized to the nucleus, compared with CAS162 reporter-expressing, CD26-negative MSTO cells (Fig. 2A, 5F). Furthermore, in CAS162 reporter-expressing, CD26-positive JMN cells, YS110 treatment induced a significant decrease in the relative luciferase activity (Fig. 5G). These data suggest that the CD26-CAS162 interaction negatively regulates POLR2A gene expression.

### Nuclear Translocation of CD26 Induced by Treatment with YS110 and 1F7 Suppresses POLR2A Expression in Cultured Cancer Cells and a Xenograft Model for Malignant Mesothelioma

**Figure 6:**
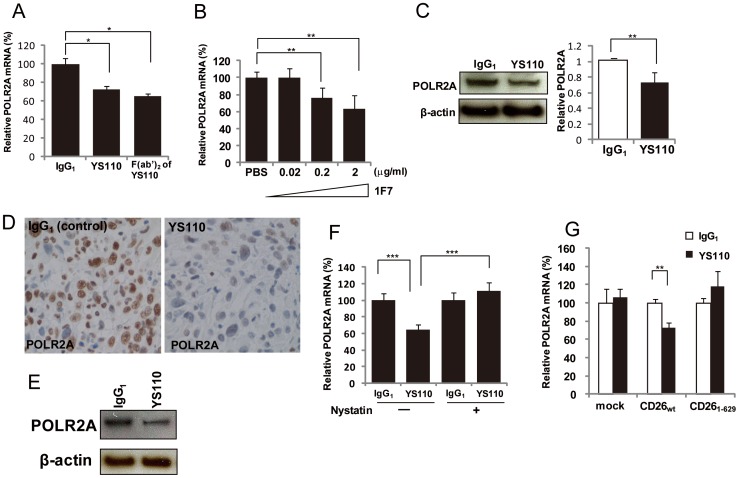
Nuclear Translocation of CD26 After Treatment with YS110 or 1F7 Suppresses POLR2A Expression. (A) Quantitative RT-PCR analysis of POLR2A mRNA in JMN cells treated with YS110 or YS110-F(ab’)_2_ (2 µg/mL) for 3 hours, relative to that in JMN cells treated with control human IgG_1_. Data were normalized to glyceraldehyde-3-phosphate dehydrogenase (GAPDH) mRNA levels and are means ± SD from three independent experiments. *P<0.01. (B) Quantitative RT-PCR analysis of POLR2A mRNA in JMN cells treated with 1F7 (0.02, 0.2, or 2 µg/mL) for 3 hours, relative to that in JMN cells treated with IgG_1_. Data were normalized to GAPDH mRNA levels and are means ± SD from three independent experiments. **P<0.025. (C) Upper panels show immunoblot analysis of POLR2A and β-actin (loading control) in lysates of JMN cells treated with control IgG_1_ or YS110 (2 µg/mL) for 3 hours. Lower panel shows mean values (± SD) from three independent experiments, for intensity of the POLR2A band in cells treated with YS110, relative to that in cells treated with control IgG_1_. (D) Immunostaining for POLR2A of tumors from NOG mice inoculated with JMN cells, followed by one intratumoral injection of control IgG_1_ or YS110 (1 µg/a tumor, volume is 100 µL). Scale bars, 20 µm. (E) Immunoblot of POLR2A and β-actin (loading control) in lysates of tumors from NOG mice inoculated with JMN cells, followed by one intratumoral injection of control IgG_1_ or YS110 (1 µg/a tumor, volume is 100 µL). (F) Quantitative RT-PCR analysis of *POLR2A* mRNA in JMN cells treated with human control IgG_1_ or YS110 (2 µg/mL) for 3 hours, after pretreatment with DMSO or nystatin (50 µg/mL) for 30 minutes. Data were normalized to GAPDH mRNA levels and are means ± SD from three independent experiments. ***P<0.005. (G) Quantitative RT-PCR analysis of POLR2A mRNA in Li7 cells transfected with control, CD26_wt_, or CD26_1–629_ constructs, following treatment with human control IgG_1_ or YS110 (2 µg/mL) for 3 hours. Data were normalized to GAPDH mRNA levels and are means ± SD from three independent experiments. **P<0.025.

To investigate whether the nuclear translocation of CD26 suppresses POLR2A expression, we first evaluated POLR2A expression in cancer cells after YS110 treatment, as YS110 induced the nuclear localization of CD26 (Fig. 2C). Quantitative reverse transcription polymerase chain reaction (qRT-PCR) analysis for POLR2A mRNA showed a significant reduction in POLR2A expression in JMN cells after treatment with YS110 or 1F7, compared with controls (Fig. 6A, 6B; Fig. S9A). Concomitantly, Western blot analysis also revealed that YS110 treatment decreased the levels of POLR2A protein in the cells (Fig. 6C). Similar results were observed in MSTO/CD26 cells treated with YS110 (Fig. S9B). Furthermore, reduced expression of POLR2A was observed in the JMN xenograft model after YS110 administration (Fig. 6D, 6E). Thus, antitumor CD26 mAbs (YS110 and 1F7) appeared to induce suppression of POLR2A expression at both the mRNA and protein levels.

To further explore the relevance of nuclear transport of CD26 and YS110 to altered expression of POLR2A mRNA, JMN cells were challenged with nystatin prior to YS110 treatment. qRT-PCR analysis showed that YS110-induced transcriptional repression of POLR2A was significantly over-ridden by nystatin treatment (Fig. 6F). Furthermore, given that the CD26_1–629_ mutant did not enter the nucleus, Li7 cells were transfected with control vector, CD26_wt_, or CD26_1–629_, and expression of POLR2A mRNA after IgG_1_ or YS110 treatment was compared by qRT-PCR. In CD26_wt_-expressing Li7 cells, POLR2A expression was significantly decreased after YS110 treatment, compared with control (Fig. 6G). Conversely, CD26_1–629_-expressing cells exhibited apparent resistance to YS110 treatment (Fig. 6G). Taken together, these results strongly suggested that nuclear localization of CD26 induced by YS110 treatment leads to suppression of POLR2A expression.

To exclude Fc domain-dependency of this reduced POLR2A expression, YS110 lacking the Fc region was prepared by pepsinization. Pepsin digestion of YS110 leads to truncation of the Fc region to yield the F(ab’)_2_ form of YS110 (Fig. S10). Treatment with the F(ab’)_2_ form of YS110 markedly decreased the levels of POLR2A transcript in JMN cells, which was similar to the result obtained with the original form of YS110 (Fig. 6A). This indicated that the effect of YS110 on transcriptional repression of POLR2A is independent of the Fc domain of YS110.

### POLR2A Localizes to the Nucleus, and POLR2A Ablation Suppresses Cell Growth of Cultured Malignant Mesothelioma Cells

**Figure 7:**
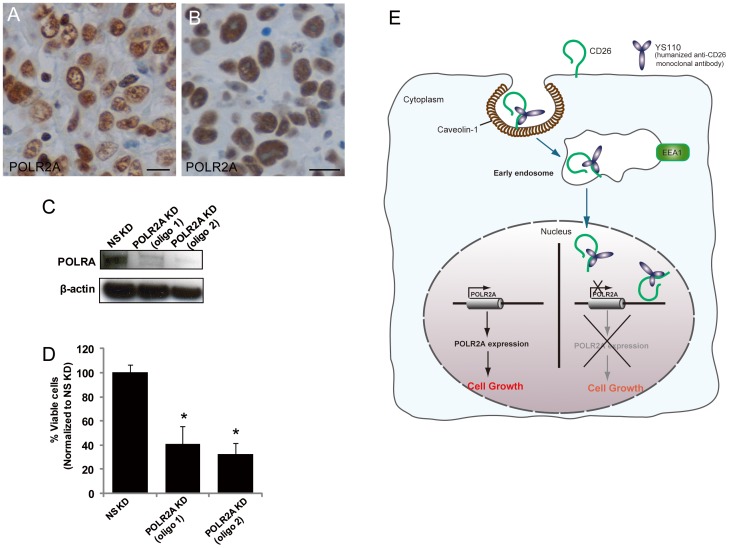
Knock-Down of the POLR2A Gene Inhibits Cell Growth in JMN Cells. (A) Immunostaining of POLR2A in tumors from malignant mesothelioma patients. Scale bar, 20 µm. (B) Immunostaining of POLR2A in tumors from NOG mice inoculated with MSTO/CD26 cells. Scale bar, 20 µm. (C) Immunoblot analysis of POLR2A in JMN cells transfected with a non-specific (NS) control siRNA or POLR2A siRNA (oligo 1 or oligo 2). β-actin was used as a loading control. KD, knock-down. (D) Numbers of viable JMN cells transfected with POLR2A siRNA (oligo 1 or oligo 2) for 48 hours, relative into the numbers of viable cells transfected with NS control siRNA, were measured using the water soluble, 2-(2-methoxy-4-nitrophenyl)-3-(4-nitrophenyl)-5-(2,4-disulfophenyl)-2H-tetrazolium (WST)-8 assay. Data are means ± SD from three independent experiments. *P<0.004. (E) Model for POLR2A suppression by YS110-induced nuclear CD26. Cell surface CD26 is translocated to the nucleus in response to YS110 treatment, and binds to genomic DNA associated with the POLR2A gene. This results in transcriptional suppression of POLR2A and consequent inhibition of cell growth.

POLR2A has been shown to be recruited to the nucleus [23]. Therefore, we examined the pattern of expression of POLR2A in tumor sections from patients with malignant mesothelioma (Fig. 7A), and in JMN (Fig. 6D) and MSTO/CD26 (Fig. 7B) cells, by immunohistochemical analysis. As previously reported for various types of tissues and tumors [36], most of the POLR2A staining was located in the nucleus in these tumors (Fig. 6D, 7A, 7B). Therefore POLR2A appears to be functional in malignant mesothelioma cells.

To examine the functional role of POLR2A in the growth of cancer cells, two RNAi constructs (oligo 1 and oligo 2) were developed to silence POLR2A expression. Both constructs effectively knocked down endogenous POLR2A in JMN cells (Fig. 7C), and obviously inhibited the proliferation of JMN cells (Fig. 7D). This result suggests that POLR2A contributes to the regulation of malignant mesothelioma cell growth.

## Discussion

Various cancer-related cell-surface proteins are known to be transported into the nucleus in several cancers, including ErbB2 (breast cancer), CD40 (lymphoma), and CD44 (breast cancer) [18], [19], [37]. The nuclear function of these receptors is implicated in critical cellular processes, from signal transduction to cell proliferation, which underscores the importance of the nuclear function of membrane receptors in cancer treatment [38]. In this study, we examined the significance of the nuclear localization of CD26 in its role as a mAb therapy for CD26-positive cancers, and showed that nuclear translocation of CD26 induced by YS110 treatment reduces cell growth through transcriptional repression of the POLR2A gene. Furthermore, we found that YS110 is itself translocated to the nucleus by a mechanism that depends on the C-terminus of CD26 (Fig. 2E; Fig. S3B). These studies highlight the function of CD26 that is translocated to the nucleus in cell growth regulation, and indicate an important mechanism by which mAb therapy targets the cell-surface antigen, CD26, that is able to translocate to the nucleus.

CD26 expression has been shown to be associated with tumor formation and metastasis [5]. We previously reported that cytoplasmic, but not cell surface, expression of CD26 in patients with malignant mesothelioma was correlated with a poor prognosis and chemo-resistance 7,8. These previous observations, together with the present findings, suggest that CD26 contributes to both tumor development and tumor growth retardation. It is possible that these reciprocal functions of CD26 in cancer cells may be determined by its functional subcellular localization, through the action of different stimuli. Therefore, we speculate that nuclear CD26 may serve as a “brake” on tumor growth. In keeping with this, other studies have shown that nuclear ErbB-2 is involved in the progression of breast carcinoma [39]. Furthermore, EGFR is known to undergo nuclear localization in regenerating hepatocytes [40]. Taken together, the nuclear translocation and the nuclear function of transmembrane proteins provide important insights into understanding the intricate cellular processes involved in development, differentiation, and tumorigenesis, and may lead to the identification of therapeutic targets for specific antigen-responsive disorders, including cancers.

The nuclear entry of most membrane proteins is thought to be mediated by mechanisms involving endocytosis and early endosomal sorting [18]. EEA1, an early endocytic protein, is known to interact with other receptors that are translocated into the nucleus, including EGFR and ErbB-2 [34], [35]. The present study showed that YS110 colocalized with EEA1, both in the cytoplasm and in the nucleus, and that transfection with a dominant-negative mutant of Rab5A, a master regulator of endosome biogenesis, prevented the nuclear translocation of YS110 (Fig. S5E). These findings suggest that early endocytic vesicles may function as carriers of cargo proteins toward the nucleus.

Furthermore, impairment of caveolin-dependent endocytosis inhibited the nuclear entry of both CD26 and YS110, and subsequent suppression of POLR2A, suggesting that the caveolin-dependent endocytic pathway is required for nuclear translocation of CD26 and YS110. Given that CD26 contains no putative nuclear localization signal (NLS) sequence, and that nuclear trafficking of CD26 and YS110 relies on the C-terminal extracellular domain of CD26 (Fig. 2E; Fig. S3B), we assume the presence of putative partners interacting with CD26. The partners presumably contain well-known nuclear accessible domains, such as NLS, and bind to CD26 through its C-terminus domain, either at the raft-domain or during endosomal trafficking associated with the early endosome. The formation of such a complex would allow full-length CD26 to pass through the nuclear membrane into the nucleus. When this interaction between the putative partners and CD26 is disrupted, the import of CD26 from the cytoplasm into the nucleus is impaired, resulting in a loss of function of nuclear CD26. Therefore, spatial expression of the putative partners may be an indispensable and rate-limiting step in both the nuclear transport and function of CD26, and this may provide one explanation for the small amount of nuclear CD26, even after YS110 treatment. In support of this, the present data showed that YS110-induced nuclear induction of CD26 did not occur in some non-neoplastic cell lines, such as HEK293 cells transfected with full-length CD26, and normal human T lymphocytes with expression of CD26 on the cell surface and in the nucleus (data not shown).

RNA polymerase II is essential for the transcription of most protein-coding genes, including those related to cell proliferation [23]. It was previously reported that blockade of POLR2A function by RNAi strategies, and treatment with chemical compounds such as α-amanitin, resulted in growth inhibition of cancer cells [41], [42], [43]. In this study, we showed that nuclear accumulation of CD26 promoted POLR2A suppression, leading to a reduction in cell growth (Fig. 7E). Taken together, these results suggest that POLR2A may provide a meaningful therapeutic target for cancers. However, we did not identify the essential region of CD26 that is required for association with DNA, and this requires further investigation.

Most therapeutic mAbs are thought to alter signal transduction within tumor cells or eliminate critical cell-surface antigens [11]. These effects may lead to the consequent clearance of cancer cells. ErbB2 is known to associate with a specific locus on the cyclooxygenase (COX) 2 promoter, activate expression of the gene, and thereby promote cell growth [39]. The humanized ErbB2 mAb trastuzumab (Herceptin) impairs the translocation of ErbB2 into the nucleus. The present study revealed that in contrast to this ErbB2-Herceptin axis, YS110 treatment abundantly augments nuclear localization of CD26, and consequently suppresses POLR2A expression, leading to inhibition of malignant mesothelioma cell growth. These findings suggest that alteration of nuclear transport of cell-surface antigens by mAbs may represent an effective target for mAb therapy of cancers.

Almost all patients with malignant mesothelioma cases have positive CD26 [7]. In this study, we showed the nuclear localization of CD26 in the samples taken from malignant mesothelioma patients. However, whether the extent of the nuclear localization is dependent on cancer types, tumor stages, and milieu or not needs further investigation. Currently, phase 1 clinical trial of YS100 treatment is conducting for malignant mesothelioma and renal cell carcinoma patients in France. The mechanism of anti-tumor machinery for YS100 and the current chemotherapeutic agents is different. Thus, combination between YS110 and standard therapy may have synergistic effects and enhance the treatment against malignant mesothelioma.

Recent studies have shown that various mAbs conjugated to payloads (e.g., radioisotopes, drugs, or toxins) may be targeted to directly kill tumor cells [44], [45], [46], [47]. In fact, the 90Y-radiolabeled murine anti-CD20 IgG_1_, Ibritumomab tiuxetan (Zevalin), has been proven to have substantial antitumor activity, and is used in standard clinical practice, as a therapy for lymphoma [48]. However, the potentially potent cytotoxicity of these payloads may impede the development of new conjugated antibodies. In this study, we demonstrated nuclear localization of anti-CD26 mAbs (YS110 and 1F7) in a cell-surface CD26-dependent manner. This implies that YS110 and 1F7 may target specific intra-nuclear components, such as genomic DNA and transcription factors. In addition to these antibodies, there have been previous reports on the nuclear localization of mAbs directed against cell-surface antigens, including ME425 (against EGF receptor) and Br 15-6A (carbohydrate Y determinant) [49], [50]. Therefore, these observations provide an insight into the development of bispecific mAbs that more potently and effectively target nuclear components related to cancer growth and invasion.

In conclusion, the present data provides evidence that induced nuclear localization of CD26 by the humanized anti-CD26 mAb, YS110, promotes transcriptional repression of the POLR2A gene, resulting in growth suppression of cancer cells. Given that YS110 has a direct anti-proliferative effect on cancer cells, including malignant mesothelioma cells, these findings highlight the potential of rational therapy against CD26-positive cancers, not only through immunological ADCC and complementary activation effects, but also by direct inhibition of cancer cell growth.

## Supporting Information

Figure S1 **Inhibition of Cell Growth by YS110 Treatment in Cultured Cancer Cells.** (A) JMN or MSTO cells were cultured overnight at a density of 2.5×10^3^ cells/well in 96-well plates. Proliferation was measured 48 hours after treatment with YS110 at the indicated concentrations, in triplicate for each condition, using cell counting reagent, as described in the MATERIALS AND METHODS. The ratio of growth inhibition was calculated as the percentage reduction in absorbance of cells treated with YS110, relative to that in cells not treated with YS110. Data are means ± SD from three independent experiments. * P<0.025. (B) T cell lymphoma Karpas299 cells were cultured overnight at a density of 1×10^4^ cells/well in 96-well plates. Proliferation was measured 24 hours after treatment with murine control mouse IgG_1_ or 1F7 (2 µg/mL), in triplicate for each condition, using cell counting reagent, as described in the MATERIALS AND METHODS. The cell viability ratio was calculated as the percentage absorbance of cells treated with 1F7 relative to that of cells treated with IgG_1_. Data are means ± SD from three independent experiments. * P<0.025.
(TIF)

Figure S2 **Nuclear Localization of Various CD26 Constructs in Several Cancer Cell Lines.** (A) Jurkat/CD26 cells treated with mouse control IgG_1_ or 1F7 (2 µg/mL) for 1 hour were fractionated into membrane, cytoplasmic, and nuclear fractions, as described in the MATERIALS AND METHODS. Each fraction was subjected to immunoblot analysis with antibody to CD26. Nuc, nuclear fraction. Mem, membrane fraction. (B) Hepatocellular carcinoma Li7 cells transiently expressing each flag-tagged construct were treated with control IgG_1_ or YS110 (2 µg/mL) for 3 hours, then subjected to subcellular fractionation, followed by immunoblot analysis with antibodies to Flag, Na^+^/K^+^ ATPase (as a cytosolic marker), and lamin A/C (as a nuclear marker). Nuc, nuclear fraction. Mem, membrane fraction.
(TIF)

Figure S3 **Nuclear Observation of Various CD26 Constructs in Several Cancer Cell Lines.** (A) Confocal visualization of GFP-CD26_wt_, GFP-CD26_7–766_, and GFP-CD26_1–629_ in HEK 293 cells, treated or not treated with Alexa-YS110 for 5 minutes. Co-localization of GFP-CD26_1–629_ with YS110 (red) appears as yellow. Scale bars, 10 µm. (B) Confocal visualization of GFP-CD26_wt_ and GFP-CD26_1–629_ in JMN cells incubated with or without Alexa-YS110 (2 µg/mL) for 30 minutes before fixation. Each GFP is shown in green, YS110 is shown in red, and the nucleus is shown in blue (Hoechst 33342). Co-localization of GFP-CD26_wt_ and YS110 in the nucleus appears as white in the boxed region. Scale bars, 10 µm.
(TIF)

Figure S4 **Nuclear Transport of CD26 Constructs Preferentially Expressed at the Cell-Surface in Jurkat/CD26 Cells.** (A) Cell surface proteins on Jurkat/CD26 cells were biotinylated using NHS-biotin, treated with control IgG_1_ or 1F7 (2 µg/mL) for the indicated times, and then fractionated into three cellular fractions. Extracts of each fraction were immunoprecipitated with antibody to CD26, and subjected to immunoblot analysis using streptavidin. The relative intensities of the streptavidin bands in the nuclear (left panels) and membrane (right panels) fractions were assessed by densitometry. Data are means ± SD from three independent experiments. Nuc, nuclear fraction. Mem, membrane fraction. (B) Cell surface-biotinylated Jurkat/CD26 cells were treated with control IgG_1_, 1F7, or 5F8 (2 µg/mL) for 1 hour before subcellular fractionation. Extracts of the membrane and nuclear fractions were immunoprecipitated with CD26 and subjected to immunoblot analysis with streptavidin. A representative immunoblot and the corresponding quantification are shown.
(TIF)

Figure S5 **Involvement of the Caveolin-Dependent Endocytic Pathway in the Nuclear Localization of YS110.** (A) JMN cells were treated with both Alexa-YS110 and Alexa-CtxB (2 µg/mL) for 10 or 30 minutes, fixed, and then stained with Hoechst 33342. The interaction of Alexa-YS110 and Alexa-CtxB (boxed regions) is demonstrated at higher magnification in the medium size images. Scale bars, 10 µm. (B) JMN cells were pretreated with chlorpromazine, an inhibitor for clathrin pathway, (10 µg/mL) for 30 minutes prior to treatment with Alexa-YS110 for 30 min. Endocytosis and nuclear localization of Alexa-YS110 (arrows) were observed by confocal fluorescence microscopy. (C) Immunofluorescence staining for YS110 (red), early endosome antigen (EEA) 1 (green), and Hoechst 33342 (blue) in fixed JMN cells, following treatment with Alexa-YS110 for 10 or 30 minutes. The boxed region in the panel shows co-localization of Alexa-YS110 with EEA1 in the nucleus (white) at high magnification. Scale bars, 10 µm. (D) Immunoelectron microscopic examination showed co-localization of EEA1 and YS110 in the nucleus of JMN cells. The arrow and arrowhead indicate EEA1 (15 nm) and YS110 (30 nm), respectively. Scale bar, 200 nm. Cy, cytoplasm; Nu, nucleus. (E) JMN cells were transfected with GFP-Rab5A^wt^ or GFP-Rab5A^S34N^. Each transfectant was treated with Alexa-YS110 for 30 minutes, fixed, then stained with Hoechst 33342. Localization of Alexa-YS110 (red) in the nucleus (blue) is indicated by arrows. Scale bars, 10 µm.
(TIF)

Figure S6 **Inhibition of Tumor Growth by YS110 Treatment in a Malignant Mesothelioma Xenograft Model.** Macroscopic images of tumors on chest walls that were developed in NOG mice orthotopically inoculated with MSTO/CD26 cells, after injection of control IgG_1_, or YS110 (left image) in thoraxes. Right panels indicate the tumor weights of left thoraxes and pericardiums in mice orthotopically inoculated with MSTO/wt or MSTO/clone12 cells, after injection of control IgG_1_ or YS110 in right thoraxes.
(TIF)

Figure S7 **YS110 is Translocated to the Nucleus in Malignant Mesothelioma Tumors.** Fluorescence analysis of subcutaneous JMN tumors from NOG mice, 1 hour after one intratumoral injection (1 µg/a tumor, volume is 100 µL) of Alexa647-human IgG_1_ (A–C) or Alexa647-YS110 (D–F). In each image, Alexa647-labeled antibodies is shown in green (B, C, E and F), and the nucleus is shown in red (Hoechst 33342) (A, C, D and F). Localization of Alexa-YS110 in the nucleus is shown as yellow (arrows) (F). Similar results were obtained with three different mice. Scale bars, 10 µm.
(TIF)

Figure S8 **ChIP Assay Using Different Primers for CAS162 in JMN cells and Reporter Assays in Various Cancer Cell Lines.** (A) The interaction between CD26 and CAS162 was detected by ChIP assay using two different types of primers flanking CAS162. Results obtained using primer set #1 are shown in Figure 5C. (B) Hepatocellular carcinoma cell lines (Li7 without CD26 expression and Kim1 with CD26 expression) was co-transfected with pGL3 promoter vector (pGL3pro, as control) or pGL3 promoter-CAS162 vector (CAS162) and phRL-TK vector, and relative luciferase activity was measured using a luminometer. Data were normalized for luciferase activity in cells transfected with phRL-TK.
(TIF)

Figure S9 **Quantitative RT-PCR Analysis of POLR2A in JMN and MSTO/CD26 Cells Treated with YS110, and Nuclear Localization of YS110-F(ab’)_2_.** (A) Quantitative RT-PCR analysis of POLR2A mRNA in JMN cells treated with YS110 (2 µg/mL), at the indicated times (1, 3, 6, 12 and 24 hours), relative to the 0 hour control. Data were normalized to the expression of glyceraldehyde-3-phosphate dehydrogenase (GAPDH) mRNA and are means ± SD from three independent experiments. *P<0.025. **P<0.006. (B) Quantitative RT-PCR analysis of POLR2A mRNA in MSTO/CD26 cells treated with control IgG_1_ or YS110 (2 µg/mL) for 3 hours. Data were normalized to the expression of GAPDH mRNA and are means ± SD from three independent experiments. *P<0.025.
(TIF)

Figure S10 **Nuclear Localization of YS110-F(ab’)_2_.** Immunoblot detection of YS110-F(ab’)_2_ in cytoplasmic and nuclear fractions of JMN or Alex (hepatocellular carcinoma) cells treated with YS110-F(ab’)_2_ (2 µg/mL) for 1 hour.
(TIF)
