# Supplementary figures and images for: Nuclear Localization of CD26 Induced by a Humanized Monoclonal Antibody Inhibits Tumor Cell Growth by Modulating of POLR2A Transcription
Source: PLoS One. 2013 Apr 29;8(4):e62304. doi: 10.1371/journal.pone.0062304 (PMC3639274; doi:10.1371/journal.pone.0062304)

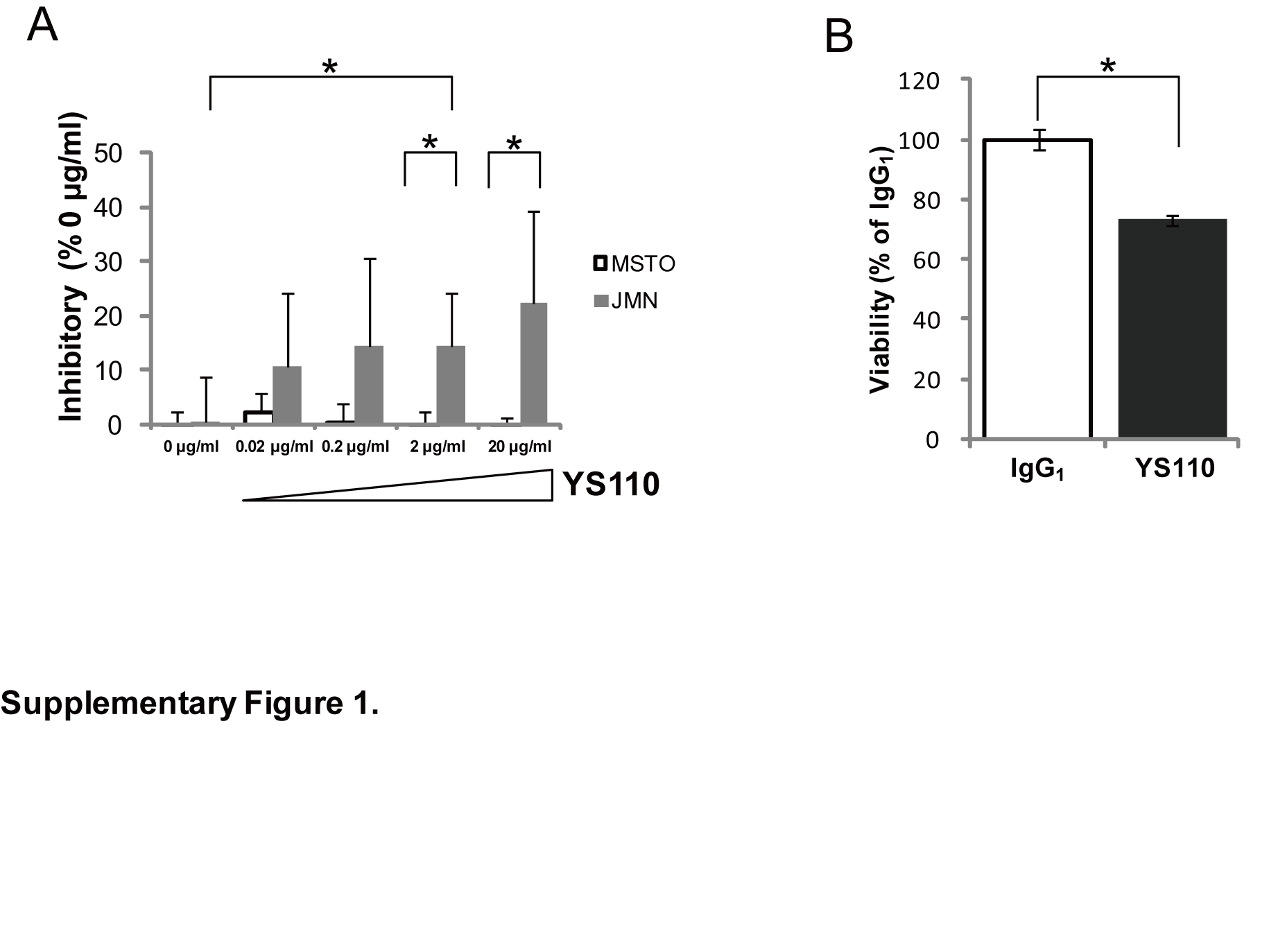

Supplement: Figure S1 — Inhibition of Cell Growth by YS110 Treatment in Cultured Cancer Cells. (A) JMN or MSTO cells were cultured overnight at a density of 2.5×10^3^ cells/well in 96-well plates. Proliferation was measured 48 hours after treatment with YS110 at the indicated concentrations, in triplicate for each condition, using cell counting reagent, as described in the MATERIALS AND METHODS. The ratio of growth inhibition was calculated as the percentage reduction in absorbance of cells treated with YS110, relative to that in cells not treated with YS110. Data are means ± SD from three independent experiments. * P<0.025. (B) T cell lymphoma Karpas299 cells were cultured overnight at a density of 1×10^4^ cells/well in 96-well plates. Proliferation was measured 24 hours after treatment with murine control mouse IgG1 or 1F7 (2 µg/mL), in triplicate for each condition, using cell counting reagent, as described in the MATERIALS AND METHODS. The cell viability ratio was calculated as the percentage absorbance of cells treated with 1F7 relative to that of cells treated with IgG1. Data are means ± SD from three independent experiments. * P<0.025. (TIF) [file pone.0062304.s001.tif]

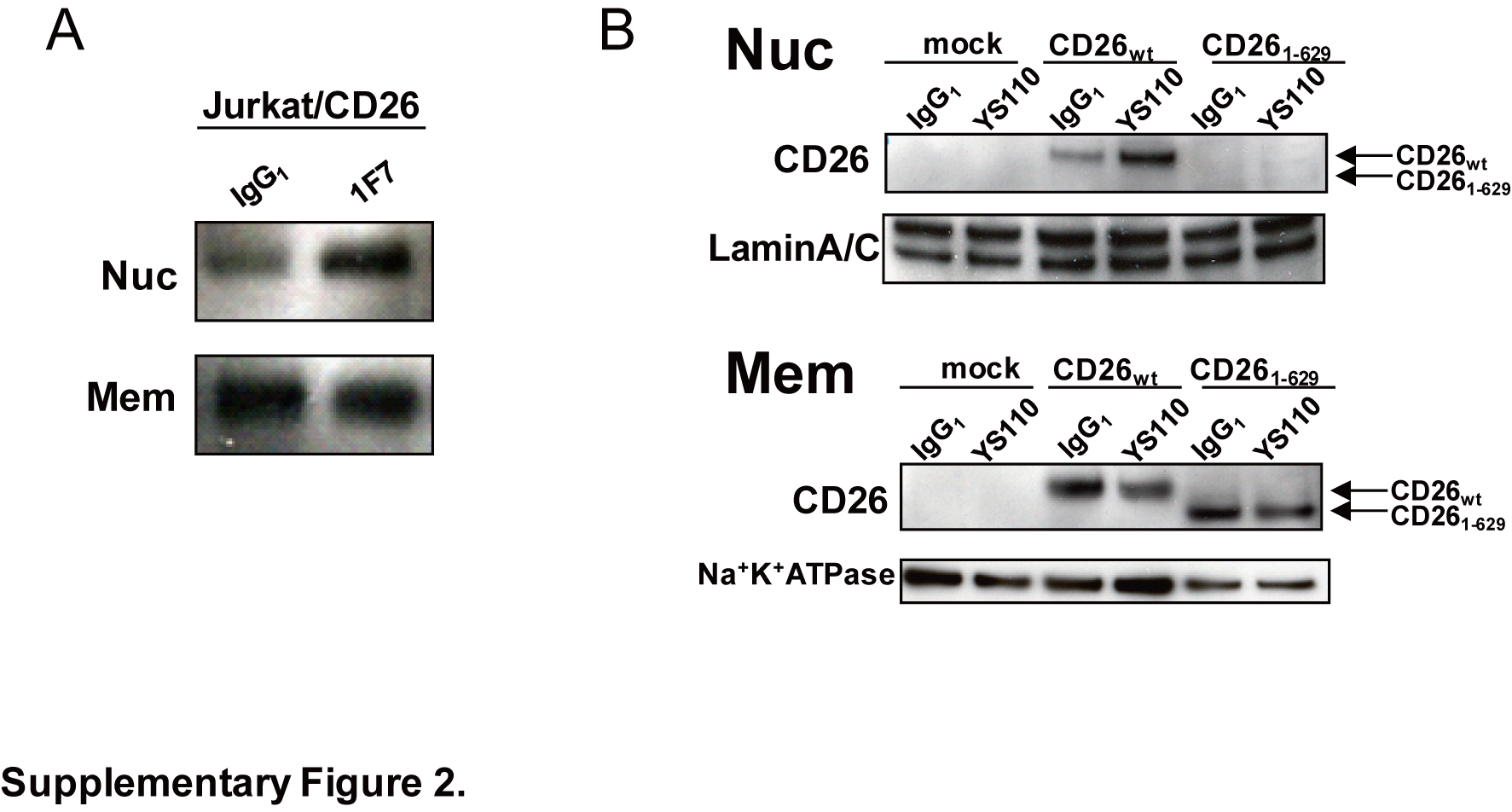

Supplement: Figure S2 — Nuclear Localization of Various CD26 Constructs in Several Cancer Cell Lines. (A) Jurkat/CD26 cells treated with mouse control IgG1 or 1F7 (2 µg/mL) for 1 hour were fractionated into membrane, cytoplasmic, and nuclear fractions, as described in the MATERIALS AND METHODS. Each fraction was subjected to immunoblot analysis with antibody to CD26. Nuc, nuclear fraction. Mem, membrane fraction. (B) Hepatocellular carcinoma Li7 cells transiently expressing each flag-tagged construct were treated with control IgG1 or YS110 (2 µg/mL) for 3 hours, then subjected to subcellular fractionation, followed by immunoblot analysis with antibodies to Flag, Na+/K+ ATPase (as a cytosolic marker), and lamin A/C (as a nuclear marker). Nuc, nuclear fraction. Mem, membrane fraction. (TIF) [file pone.0062304.s002.tif]

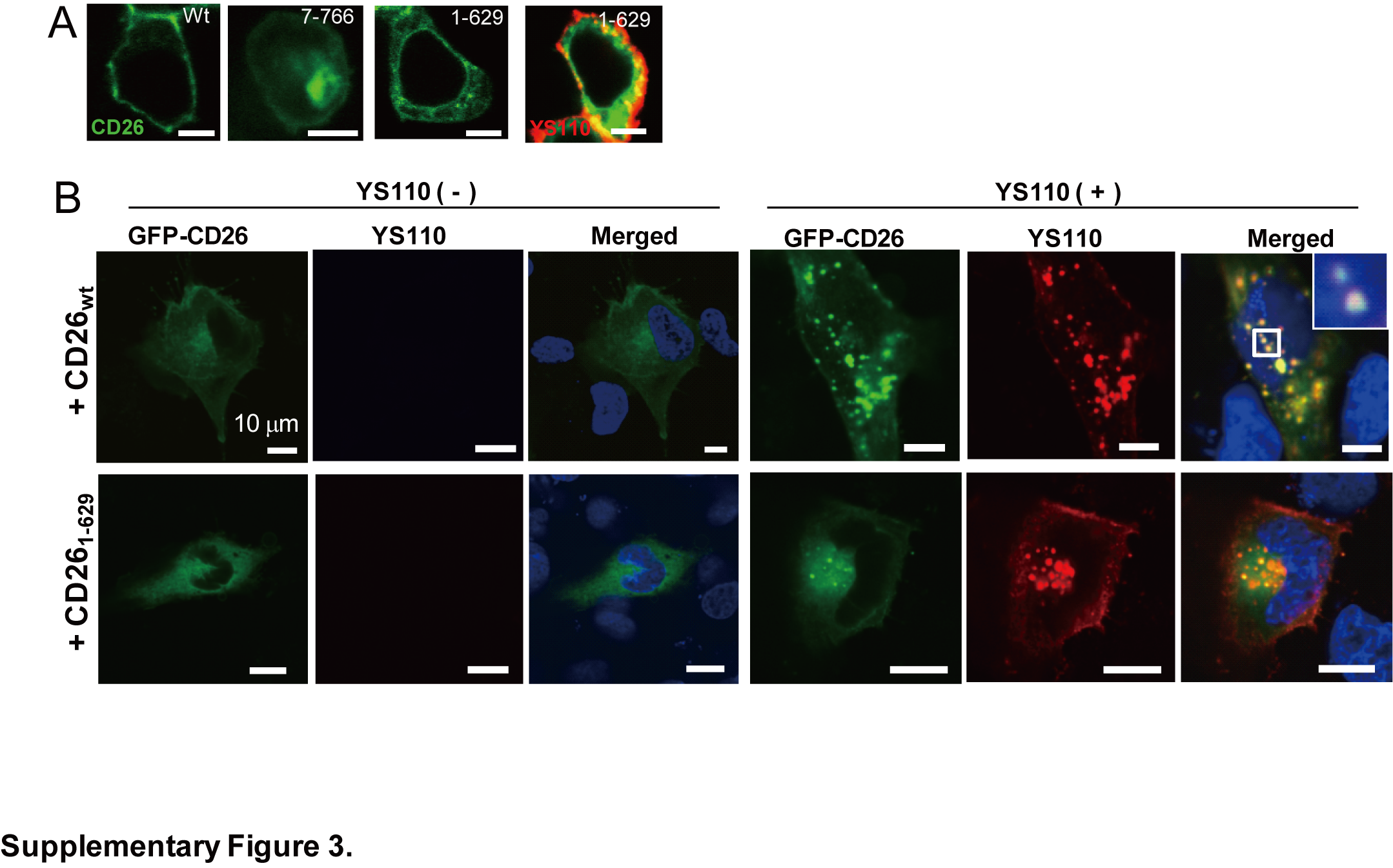

Supplement: Figure S3 — Nuclear Observation of Various CD26 Constructs in Several Cancer Cell Lines. (A) Confocal visualization of GFP-CD26wt, GFP-CD267–766, and GFP-CD261–629 in HEK 293 cells, treated or not treated with Alexa-YS110 for 5 minutes. Co-localization of GFP-CD261–629 with YS110 (red) appears as yellow. Scale bars, 10 µm. (B) Confocal visualization of GFP-CD26wt and GFP-CD261–629 in JMN cells incubated with or without Alexa-YS110 (2 µg/mL) for 30 minutes before fixation. Each GFP is shown in green, YS110 is shown in red, and the nucleus is shown in blue (Hoechst 33342). Co-localization of GFP-CD26wt and YS110 in the nucleus appears as white in the boxed region. Scale bars, 10 µm. (TIF) [file pone.0062304.s003.tif]

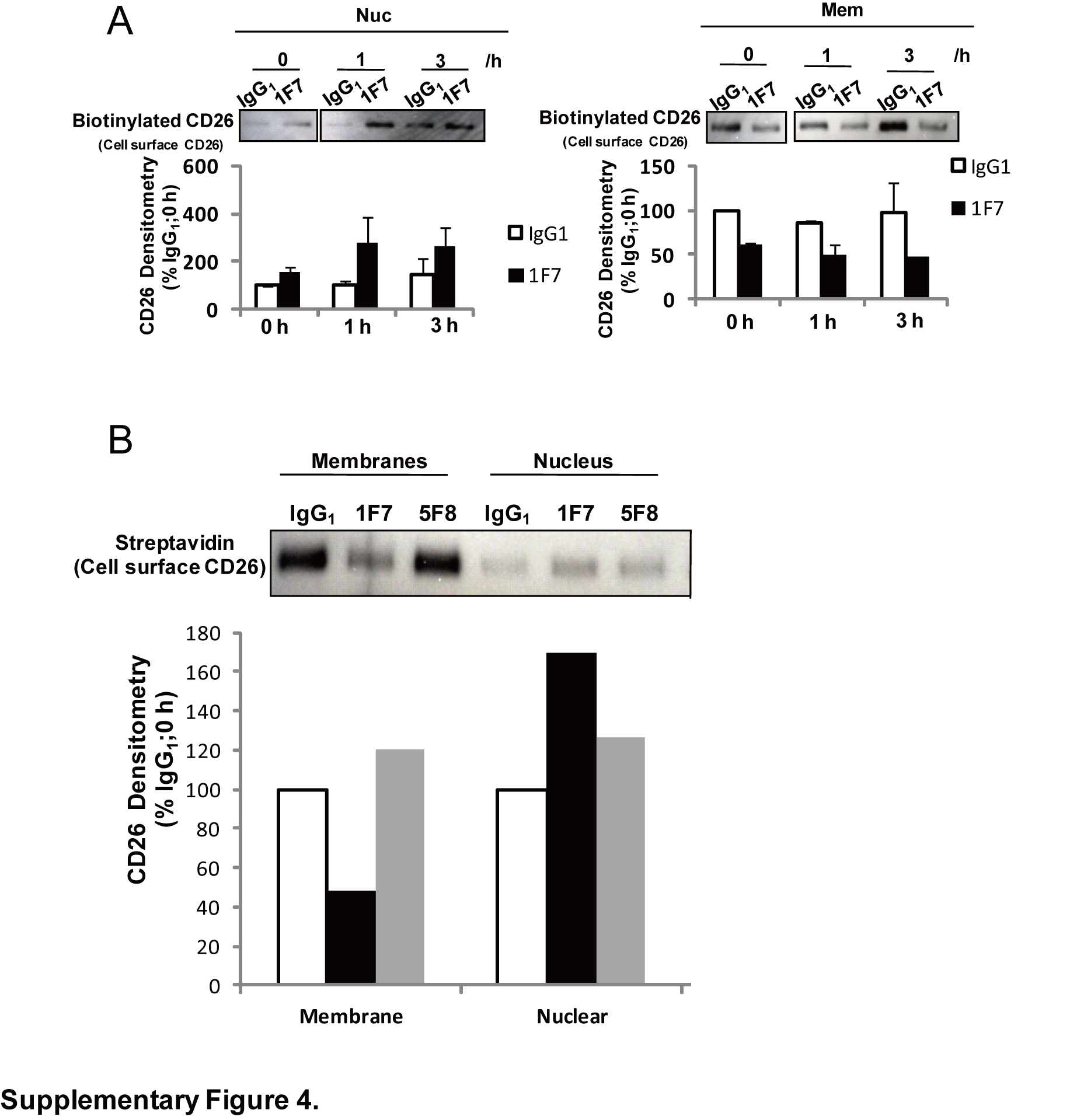

Supplement: Figure S4 — Nuclear Transport of CD26 Constructs Preferentially Expressed at the Cell-Surface in Jurkat/CD26 Cells. (A) Cell surface proteins on Jurkat/CD26 cells were biotinylated using NHS-biotin, treated with control IgG1 or 1F7 (2 µg/mL) for the indicated times, and then fractionated into three cellular fractions. Extracts of each fraction were immunoprecipitated with antibody to CD26, and subjected to immunoblot analysis using streptavidin. The relative intensities of the streptavidin bands in the nuclear (left panels) and membrane (right panels) fractions were assessed by densitometry. Data are means ± SD from three independent experiments. Nuc, nuclear fraction. Mem, membrane fraction. (B) Cell surface-biotinylated Jurkat/CD26 cells were treated with control IgG1, 1F7, or 5F8 (2 µg/mL) for 1 hour before subcellular fractionation. Extracts of the membrane and nuclear fractions were immunoprecipitated with CD26 and subjected to immunoblot analysis with streptavidin. A representative immunoblot and the corresponding quantification are shown. (TIF) [file pone.0062304.s004.tif]

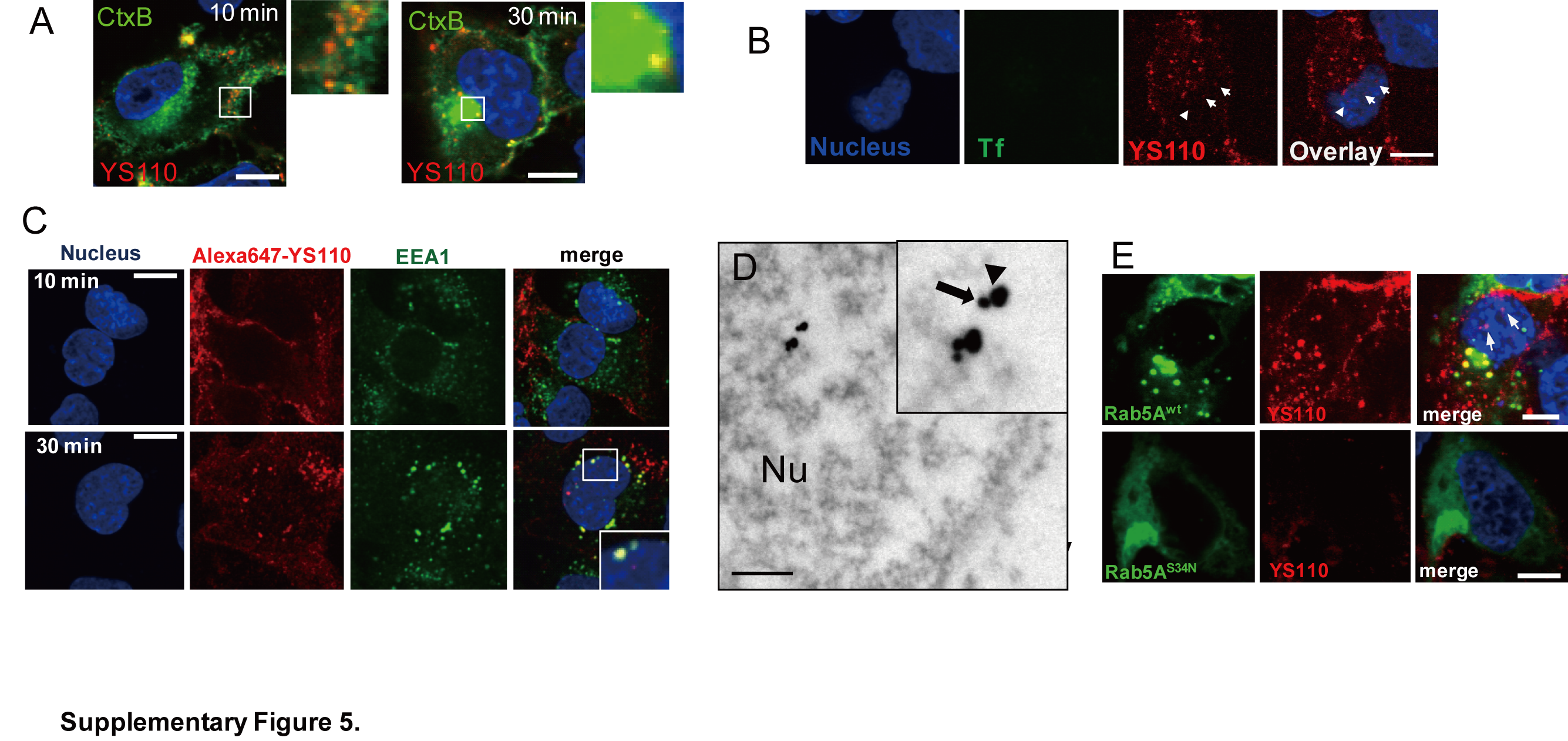

Supplement: Figure S5 — Involvement of the Caveolin-Dependent Endocytic Pathway in the Nuclear Localization of YS110. (A) JMN cells were treated with both Alexa-YS110 and Alexa-CtxB (2 µg/mL) for 10 or 30 minutes, fixed, and then stained with Hoechst 33342. The interaction of Alexa-YS110 and Alexa-CtxB (boxed regions) is demonstrated at higher magnification in the medium size images. Scale bars, 10 µm. (B) JMN cells were pretreated with chlorpromazine, an inhibitor for clathrin pathway, (10 µg/mL) for 30 minutes prior to treatment with Alexa-YS110 for 30 min. Endocytosis and nuclear localization of Alexa-YS110 (arrows) were observed by confocal fluorescence microscopy. (C) Immunofluorescence staining for YS110 (red), early endosome antigen (EEA) 1 (green), and Hoechst 33342 (blue) in fixed JMN cells, following treatment with Alexa-YS110 for 10 or 30 minutes. The boxed region in the panel shows co-localization of Alexa-YS110 with EEA1 in the nucleus (white) at high magnification. Scale bars, 10 µm. (D) Immunoelectron microscopic examination showed co-localization of EEA1 and YS110 in the nucleus of JMN cells. The arrow and arrowhead indicate EEA1 (15 nm) and YS110 (30 nm), respectively. Scale bar, 200 nm. Cy, cytoplasm; Nu, nucleus. (E) JMN cells were transfected with GFP-Rab5Awt or GFP-Rab5AS34N. Each transfectant was treated with Alexa-YS110 for 30 minutes, fixed, then stained with Hoechst 33342. Localization of Alexa-YS110 (red) in the nucleus (blue) is indicated by arrows. Scale bars, 10 µm. (TIF) [file pone.0062304.s005.tif]

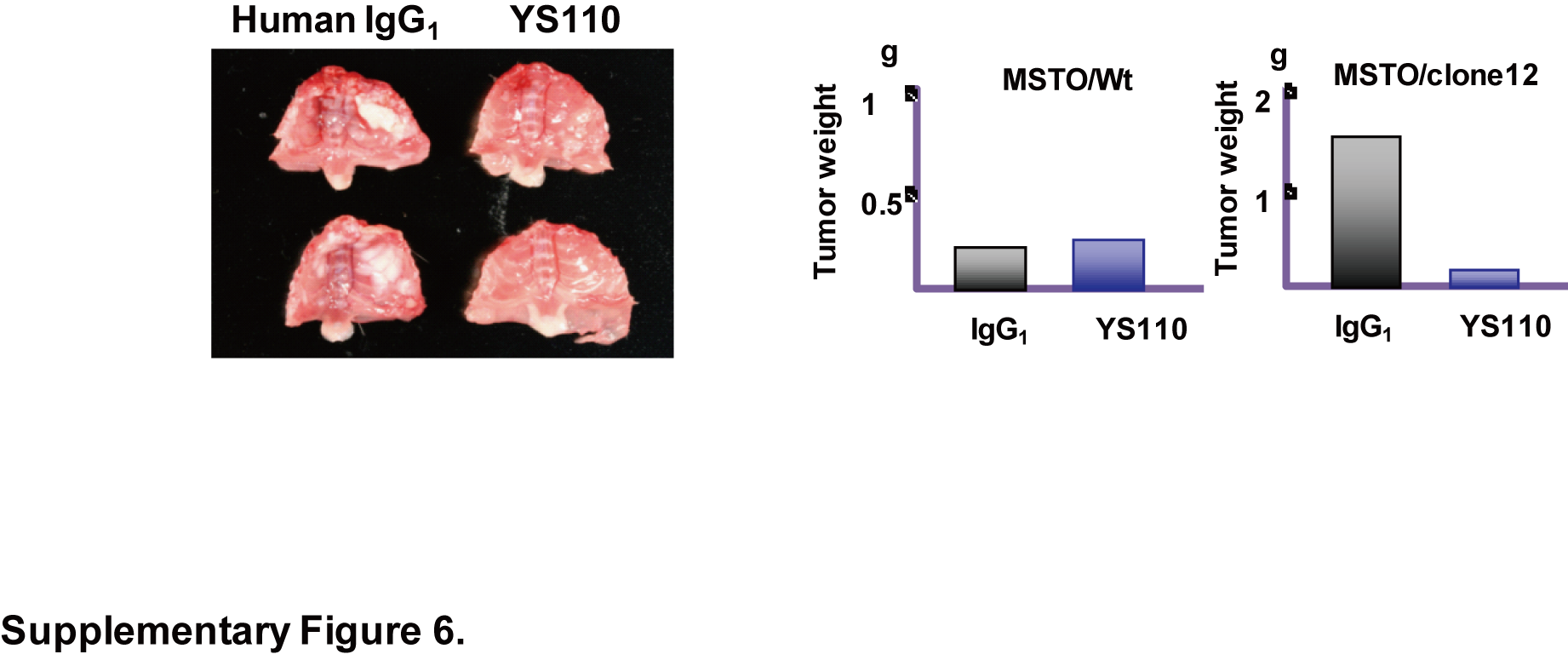

Supplement: Figure S6 — Inhibition of Tumor Growth by YS110 Treatment in a Malignant Mesothelioma Xenograft Model. Macroscopic images of tumors on chest walls that were developed in NOG mice orthotopically inoculated with MSTO/CD26 cells, after injection of control IgG1, or YS110 (left image) in thoraxes. Right panels indicate the tumor weights of left thoraxes and pericardiums in mice orthotopically inoculated with MSTO/wt or MSTO/clone12 cells, after injection of control IgG1 or YS110 in right thoraxes. (TIF) [file pone.0062304.s006.tif]

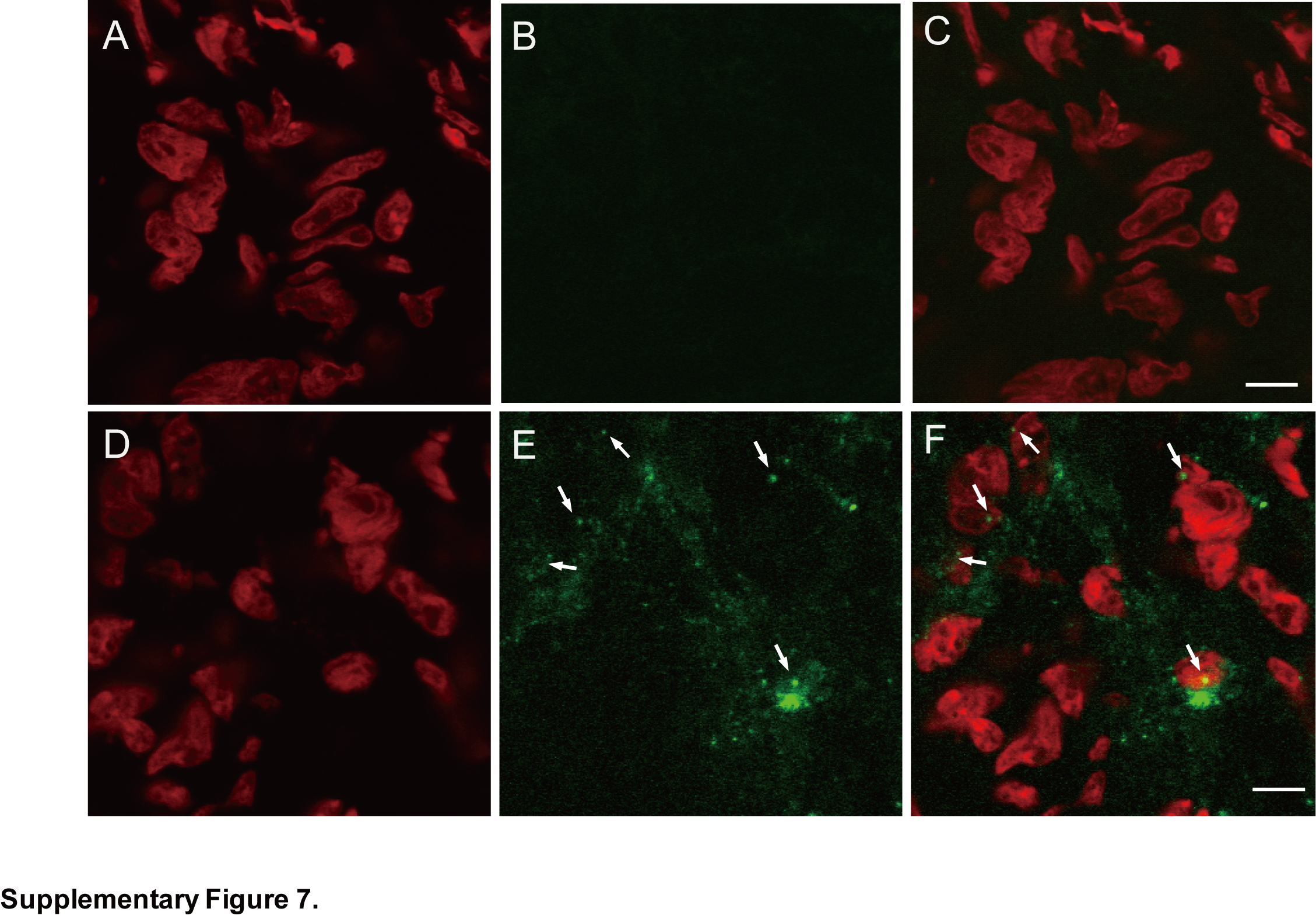

Supplement: Figure S7 — YS110 is Translocated to the Nucleus in Malignant Mesothelioma Tumors. Fluorescence analysis of subcutaneous JMN tumors from NOG mice, 1 hour after one intratumoral injection (1 µg/a tumor, volume is 100 µL) of Alexa647-human IgG1 (A–C) or Alexa647-YS110 (D–F). In each image, Alexa647-labeled antibodies is shown in green (B, C, E and F), and the nucleus is shown in red (Hoechst 33342) (A, C, D and F). Localization of Alexa-YS110 in the nucleus is shown as yellow (arrows) (F). Similar results were obtained with three different mice. Scale bars, 10 µm. (TIF) [file pone.0062304.s007.tif]

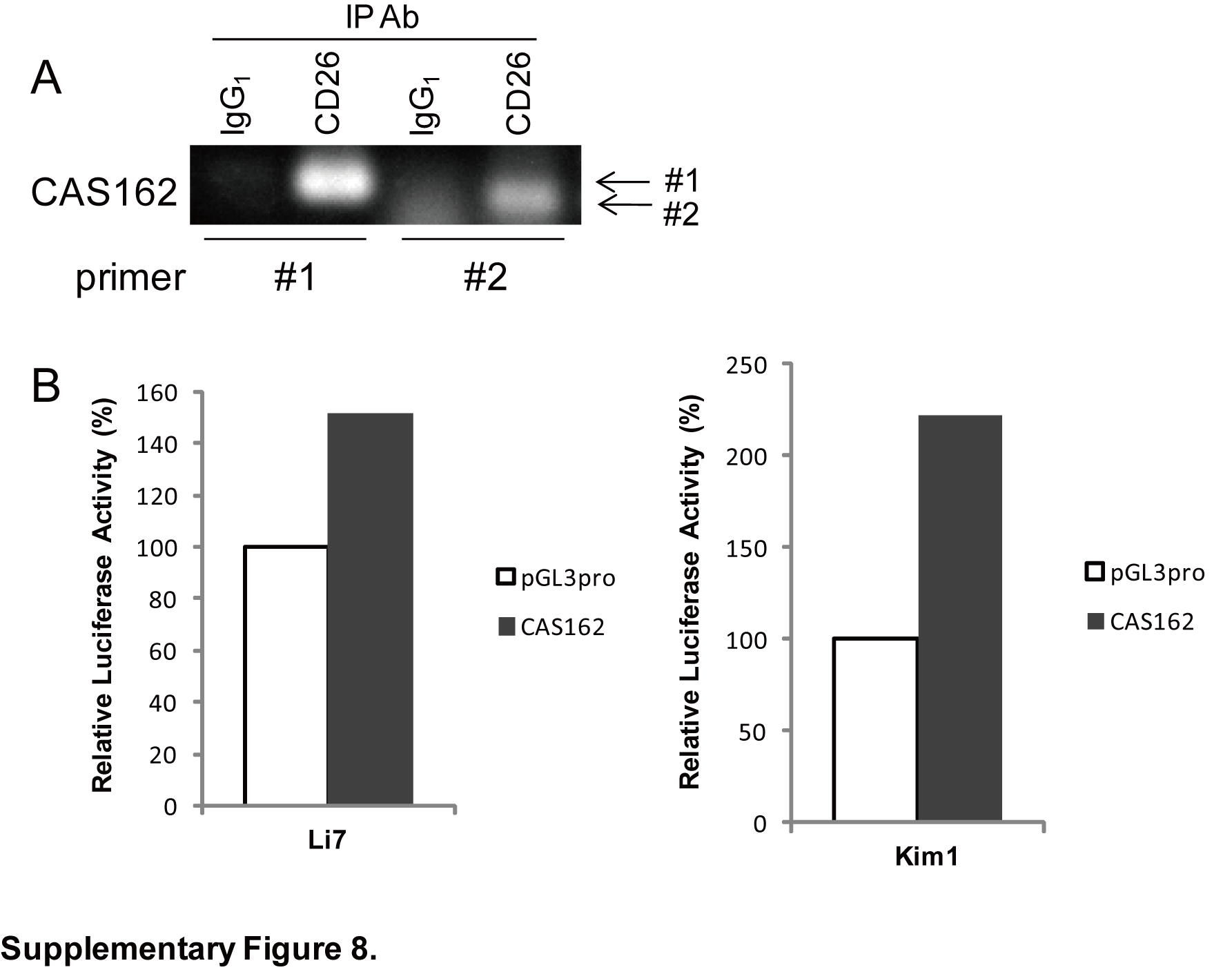

Supplement: Figure S8 — ChIP Assay Using Different Primers for CAS162 in JMN cells and Reporter Assays in Various Cancer Cell Lines. (A) The interaction between CD26 and CAS162 was detected by ChIP assay using two different types of primers flanking CAS162. Results obtained using primer set #1 are shown in Figure 5C. (B) Hepatocellular carcinoma cell lines (Li7 without CD26 expression and Kim1 with CD26 expression) was co-transfected with pGL3 promoter vector (pGL3pro, as control) or pGL3 promoter-CAS162 vector (CAS162) and phRL-TK vector, and relative luciferase activity was measured using a luminometer. Data were normalized for luciferase activity in cells transfected with phRL-TK. (TIF) [file pone.0062304.s008.tif]

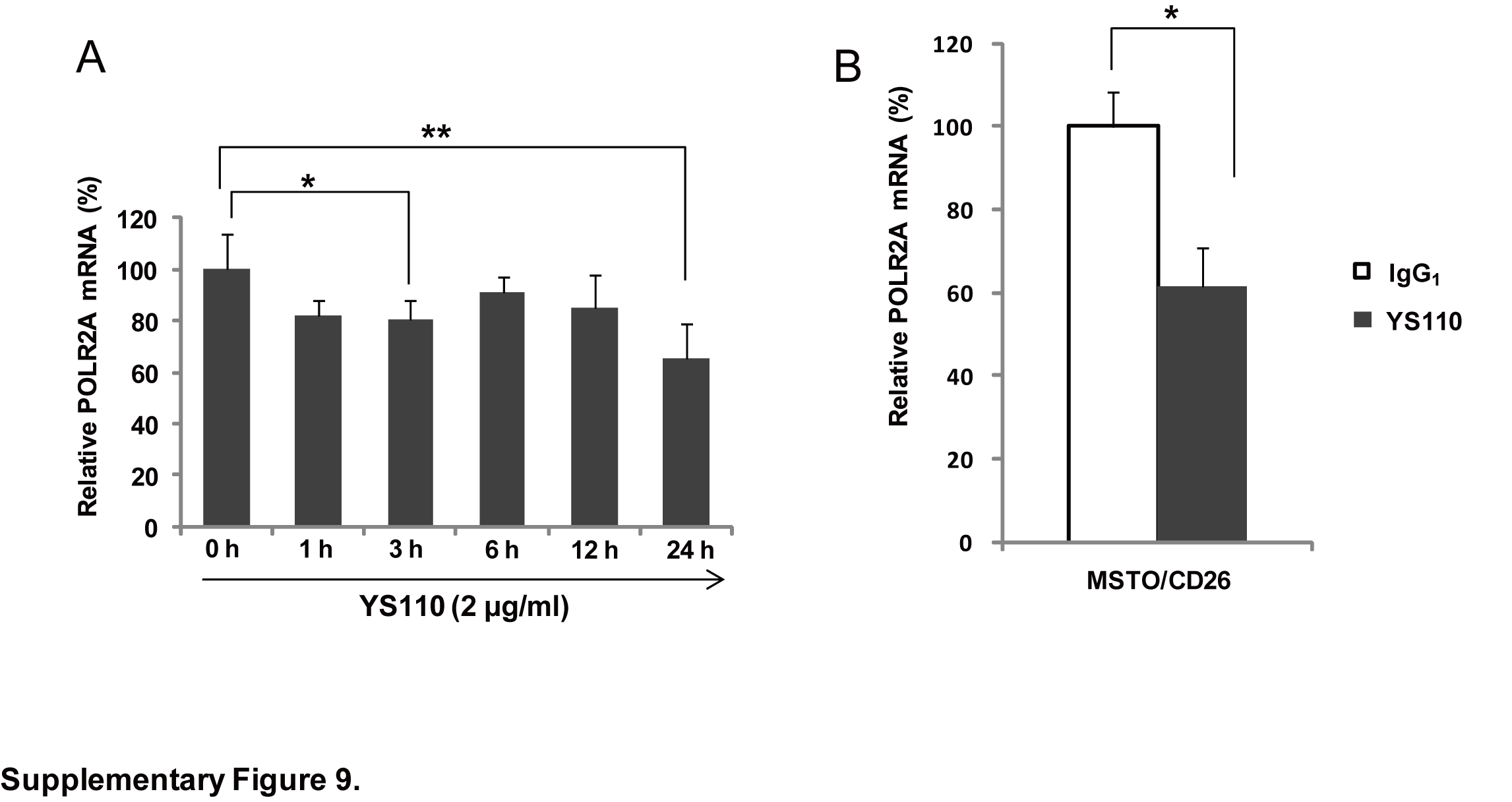

Supplement: Figure S9 — Quantitative RT-PCR Analysis of POLR2A in JMN and MSTO/CD26 Cells Treated with YS110, and Nuclear Localization of YS110-F(ab’)2. (A) Quantitative RT-PCR analysis of POLR2A mRNA in JMN cells treated with YS110 (2 µg/mL), at the indicated times (1, 3, 6, 12 and 24 hours), relative to the 0 hour control. Data were normalized to the expression of glyceraldehyde-3-phosphate dehydrogenase (GAPDH) mRNA and are means ± SD from three independent experiments. *P<0.025. **P<0.006. (B) Quantitative RT-PCR analysis of POLR2A mRNA in MSTO/CD26 cells treated with control IgG1 or YS110 (2 µg/mL) for 3 hours. Data were normalized to the expression of GAPDH mRNA and are means ± SD from three independent experiments. *P<0.025. (TIF) [file pone.0062304.s009.tif]

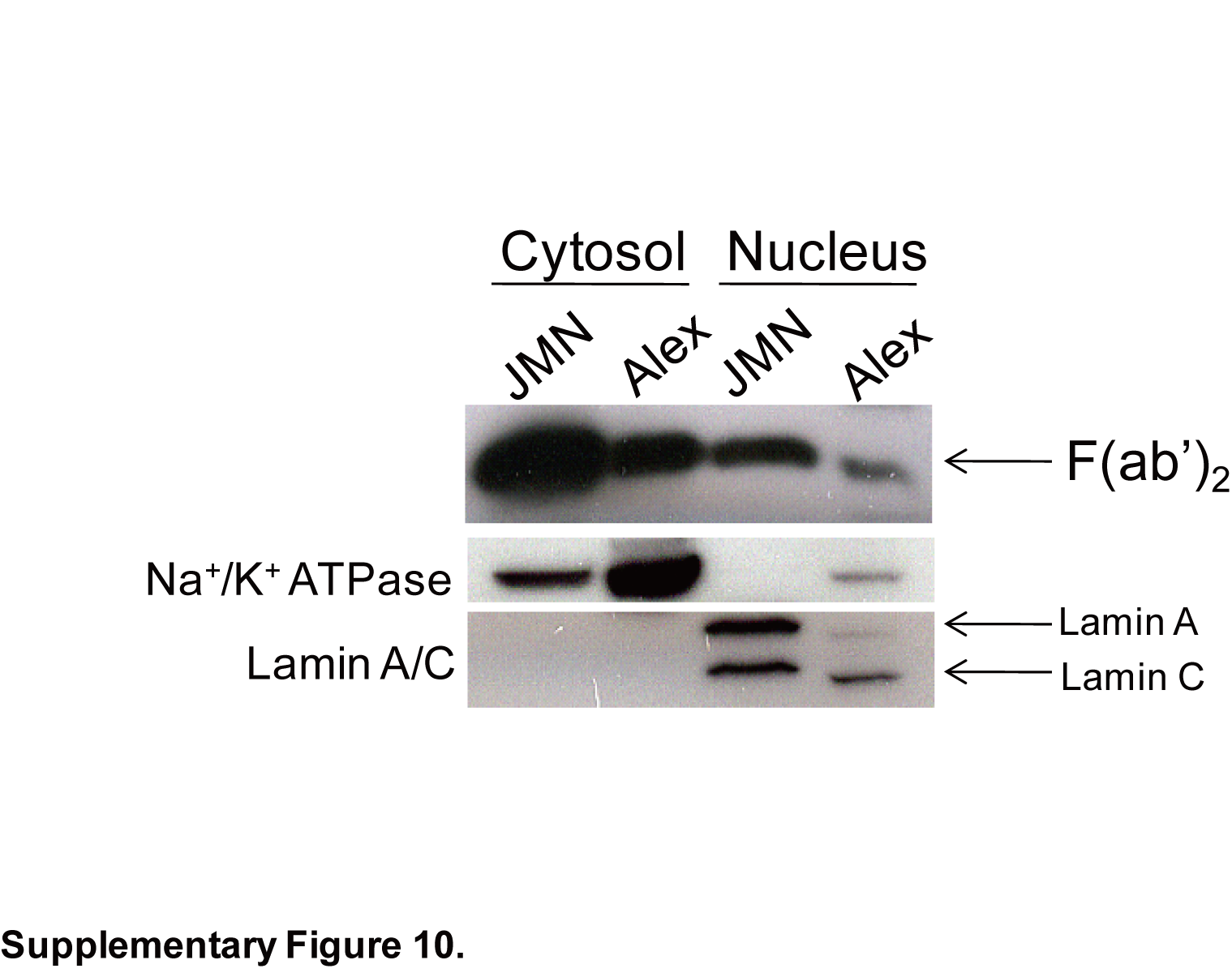

Supplement: Figure S10 — Nuclear Localization of YS110-F(ab’)2. Immunoblot detection of YS110-F(ab’)2 in cytoplasmic and nuclear fractions of JMN or Alex (hepatocellular carcinoma) cells treated with YS110-F(ab’)2 (2 µg/mL) for 1 hour. (TIF) [file pone.0062304.s010.tif]
